# Compositional and Biofunctional Properties of *Xyris* spp. and *Mimosa* spp. Bee Pollen from Thailand

**DOI:** 10.3390/foods15111990

**Published:** 2026-06-03

**Authors:** Sumed Yadoung, Pichet Praphawilai, Pichamon Yana, Peerapong Jeeno, Hataichanok Chuljerm, Khanchai Danmek, Ming-Cheng Wu, Chuleui Jung, Bajaree Chuttong, Surat Hongsibsong

**Affiliations:** 1 Environmental, Occupational Health Sciences and NCD Center of Excellence, Research Institute for Health Sciences, Chiang Mai University, Chiang Mai 50200, Thailand; sumed.yadoung@cmu.ac.th (S.Y.); pichamon.y@cmu.ac.th (P.Y.); 2 School of Health Sciences Research, Research Institute for Health Sciences, Chiang Mai University, Chiang Mai 50200, Thailand; peerapong_jeen@cmu.ac.th (P.J.); hataichanok.ch@cmu.ac.th (H.C.); 3 Office of Research Administration, Chiang Mai University, Chiang Mai 50200, Thailand; pichet.p@cmu.ac.th; 4 Meliponini and Apini Research Laboratory, Department of Entomology and Plant Pathology, Faculty of Agriculture, Chiang Mai University, Chiang Mai 50200, Thailand; 5 School of Agriculture and Natural Resources, University of Phayao, Phayao 56000, Thailand; khanchai.da@up.ac.th; 6 Department of Entomology, National Chung Hsing University, Taichung 402202, Taiwan; mcwu@nchu.edu.tw; 7 Department of Plant Medicals, Gyeongkuk National University, Andong 36729, Republic of Korea; cjung@gknu.ac.kr

**Keywords:** bee pollen, *Mimosa* spp., *Xyris* spp., antioxidant activity, FRAP, LC-MS analysis, natural product, phytochemicals

## Abstract

Methanolic bee pollen extracts from *Xyris* spp. and *Mimosa* spp. were evaluated for proximate composition, extraction yield, total phenolic content (TPC), total flavonoid content (TFC), antioxidant activity, erythrocyte oxidative hemolysis protection, HRBC membrane stabilization, and LC-QToF-MS-based phytochemical annotation. *Mimosa* spp. pollen showed higher crude protein content than *Xyris* spp. pollen (29.54% vs. 18.38%, dry basis), whereas *Xyris* spp. pollen showed higher crude fat and nitrogen-free extract. The methanolic extraction yield was higher for *Xyris* spp. than *Mimosa* spp. (44.26% vs. 34.63%). *Mimosa* spp. extract exhibited higher TPC (17.07 ± 0.19 mg GAE/g dry pollen) and TFC (8.89 ± 0.59 mg QE/g dry pollen) than *Xyris* spp. extract, which contained 10.39 ± 0.27 mg GAE/g dry pollen and 6.94 ± 0.22 mg QE/g dry pollen, respectively. *Mimosa* spp. also showed lower DPPH and ABTS IC_50_ values and higher FRAP activity. LC-QToF-MS results were reported as putatively annotated compounds based on accurate mass and database matching. These findings suggest that botanical origin influences the chemical composition and in vitro bioactivity of bee pollen; however, further targeted compound confirmation, toxicity assessment, and in vivo studies are required.

## 1. Introduction

Bee pollen, the agglomerated pollen grains collected by honey bees (*Apis mellifera*) and mixed with their salivary secretions and small amounts of nectar, has been increasingly studied as a functional natural product owing to its rich nutritional and bioactive composition [1]. It contains proteins (10–40%), carbohydrates (13–55%), lipids (1–10%), and a wide variety of secondary metabolites, particularly phenolic acids and flavonoids, which have been associated with antioxidant, anti-inflammatory, antimicrobial, and immunomodulatory activities [2,3]. However, the chemical composition and biological properties of bee pollen are not constant: they vary substantially depending on the botanical (floral) origin, geographical region, climate, and season of harvest [4,5]. Consequently, the comparative evaluation of monofloral pollens collected from different plant sources is essential to identify those with the greatest functional potential.

Although extensive work has been carried out on monofloral bee pollens from European, South American, and North African origins [4,6,7], data on bee pollens from Southeast Asia—particularly from Thailand—remain comparatively scarce. The few studies available from this region have characterized only a limited number of plant sources, leaving most of the locally relevant floral origins uncharacterized in terms of their phytochemistry and bioactivity [8,9]. Among these underexplored sources, two genera stand out: *Mimosa* spp. (Fabaceae), abundant in disturbed and upland habitats of northern Thailand, and *Xyris* spp. (Xyridaceae), characteristic of lowland swampy and paddy-field environments in the northeast. Both genera are well-known foraging resources for honey bees during the dry-cool season, when alternative floral sources are limited [9].

Despite their ecological and apicultural relevance, only a handful of studies have specifically addressed bee pollen from these two genera. For *Mimosa* spp., the most directly comparable work is that of Aragão et al. [10], who characterized monofloral *Mimosa pudica* bee pollen from northeastern Brazil and reported 62 compounds—including seven flavonoids and 15 hydroxycinnamic acid amide derivatives—together with notable DPPH and ABTS radical-scavenging activities. Earlier palynological surveys in Bahia (Brazil) also identified *M. pudica* as a dominant pollen type with phenolic contents ranging from 41.5 to 213.2 mg GAE/g [11]. In contrast, work directly relevant to Thailand is limited to the study [9], who reported the phytochemical content and antioxidant/antilipoxygenase activities of monofloral bee pollens from six Thai plant sources, including the related species *Mimosa diplotricha* and *Xyris complanata*, and identified spermidine derivatives as major bioactive constituents. For *Xyris* spp. as a bee pollen source, the published literature is even more limited, and no detailed phytochemical profile of *Xyris* spp. monofloral bee pollen—particularly from northeastern Thailand—has been reported to date. The available evidence therefore presents two clear research gaps: (i) the lack of direct comparison between *Mimosa* spp. and *Xyris* spp. monofloral bee pollens collected from their respective dominant ecological zones in Thailand, and (ii) the absence of integrated data combining proximate composition, antioxidant activity, membrane-protective effects, and untargeted phytochemical annotation by LC-QToF-MS for these two floral origins.

Addressing these gaps is relevant not only for understanding regional pollen quality, but also for evaluating the influence of botanical origin on the in vitro bioactivity of bee pollen—an aspect repeatedly emphasized as a determinant of functional value [4,5,12]. Accordingly, the present study aimed to (1) determine the proximate composition and methanolic extraction yield of monofloral bee pollen from *Mimosa* spp. and *Xyris* spp. collected in northern and northeastern Thailand; (2) evaluate their in vitro antioxidant activity (DPPH, ABTS, FRAP), total phenolic and flavonoid contents, erythrocyte oxidative hemolysis protection, and HRBC membrane stabilization; and (3) putatively annotate their phytochemical constituents by LC-QToF-MS. The findings provide preliminary in vitro evidence on the comparative bioactivity of these two underexplored Thai bee pollens; however, in vivo studies and toxicological assessments are required before any food, nutritional, or health-related applications can be considered.

## 2. Materials and Methods

### 2.1. Collection of Bee Pollen

**Figure 1:**
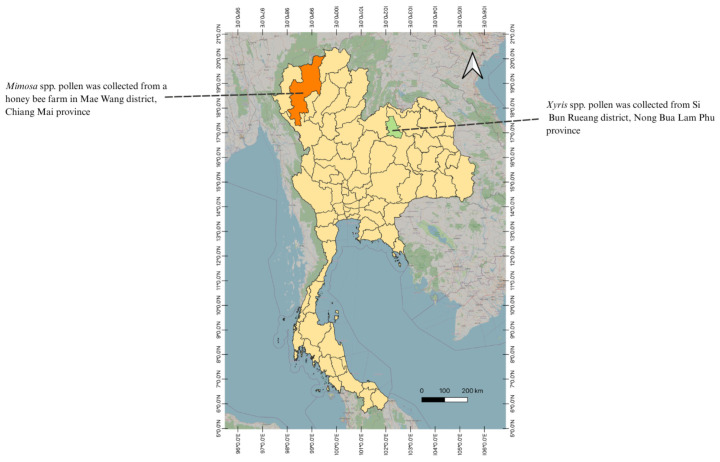
Geographical locations of the bee pollen collection sites in Thailand. *Mimosa* spp. pollen was collected from Mae Wang district, Chiang Mai province (northern Thailand; shown in orange), and *Xyris* spp. pollen was collected from Si Bun Rueang district, Nong Bua Lam Phu province (northeastern Thailand; shown in green).

Fresh pollen samples were efficiently collected using pollen traps installed on selected beehives. The monofloral pollen samples were obtained from five beehives in each location, all belonging to commercial pollen-producing farms. *Mimosa* spp. pollen was collected from a honey bee farm in Mae Wang district, Chiang Mai province, while *Xyris* spp. pollen was collected from Si Bun Rueang district, Nong Bua Lam Phu province, Thailand. The collection period spanned from October to December 2023. The geographical locations of the two sampling sites within Thailand are shown in Figure 1.

The plant sources of each pollen sample were identified according to the specific locations of the beehives, which were in areas dominated by the target plant species. Bee pollen was collected from each hive and pooled to create composite samples. Monoflorality was determined by microscopic examination, considering a plant species as dominant when its pollen grains accounted for more than 80% of the total grains observed [13].

All samples were promptly transported to the laboratory and dried at 40 °C for 48–72 h until constant weight and stored at room temperature until extraction and analysis.

**Figure 2:**
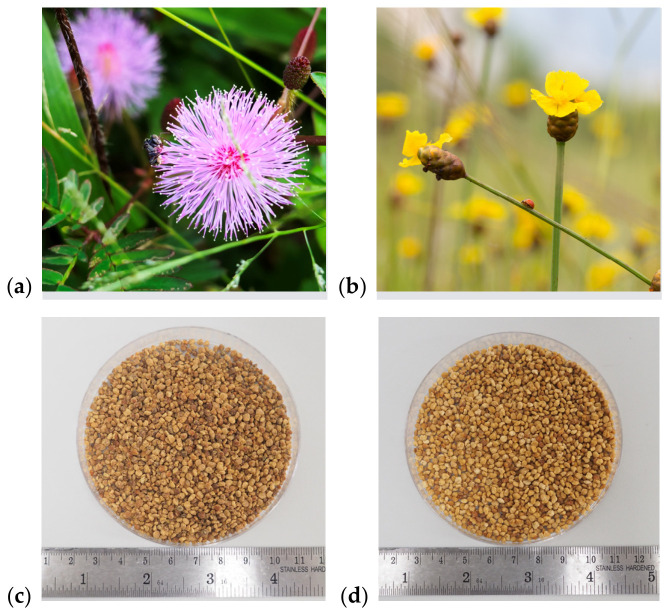
Representative images of floral sources and bee pollen pellets from *Mimosa* spp. and *Xyris* spp. (**a**) Flower of *Mimosa* spp.; (**b**) flower of *Xyris* spp.; (**c**) bee pollen pellets collected from *Mimosa* spp.; and (**d**) bee pollen pellets collected from *Xyris* spp. The floral images show the characteristic morphology of each plant source, while the pollen pellet images provide macroscopic documentation of the collected bee pollen samples before microscopic confirmation of botanical origin.

Figure 2 shows representative images of the floral sources and corresponding bee pollen pellets. *Mimosa* spp. were characterized by spherical pink inflorescences, whereas *Xyris* spp. showed small yellow flowers. The bee pollen pellets collected from these floral sources also differed in macroscopic appearance. *Mimosa* spp. pollen pellets appeared compact and yellowish-brown, while *Xyris* spp. pollen pellets showed a yellow to golden-brown coloration with visible granular texture. These macroscopic observations were used for preliminary sorting and were further supported by microscopic pollen morphology analysis.

### 2.2. Microscopy and Morphological Identification

The floral origin-based samples were prepared by visually sorting the pollen pellets according to their colors. The monofloral samples consisted of pollen with a consistent color that met the criterion of having a pollen frequency greater than 90% from a single plant taxon [9]. Pollen samples were prepared for detailed palynological analysis to identify their botanical origin. The distinctive morphological characteristics of the pollen grains, including their shape, size, and surface patterns were examined under a light microscope and scanning electron microscope (SEM) Identification was performed by comparing these characteristics to those of a pre-established pollen reference collection derived from the flowers of *Mimosa* spp. and *Xyris* spp. This comparative analysis ensured accurate identification of the monofloral pollen samples collected.

The monofloral samples were defined as those containing >90% pollen frequency from a single plant taxon [9]. Pollen identification was based on comparison of light microscopy and SEM images with a pre-established pollen reference collection prepared from the flowers of *Mimosa* spp. and *Xyris* spp.; quantitative palynological enumeration (formal pollen grain counting) was not performed in this study.

### 2.3. Bee Pollen Composition Analysis

Nutritional composition of the bee pollen samples was analyzed using a series of standardized analytical procedures [14]. Briefly, the pollen samples were ground through a 1.0 mm mesh to ensure homogeneity. The dry matter content (DM) was determined by oven-drying at 105 °C until a constant weight was achieved and calculation of moisture loss. Crude protein content (CP) was quantified using the Kjeldahl method, which involves digestion, distillation, and titration following by a conversion factor of 6.25 for estimate the protein content. Crude fat (EE) was extract using Soxhlet apparatus with petroleum ether as solvents then the solvent was removed, and the remaining fat residue was weighed to calculate the fat content. Crude fiber (CF) was assessed by sequential digestion with acid and alkali following by filtration, drying, and representing the indigestible fibrous component such as cellulose and lignin. Lastly ash content was measured by incinerating the samples in a muffle furnace at 550 °C. Then, the ash content was calculated based on the weight difference before and after ashing.

### 2.4. Bee Pollen Extraction

Dried bee pollen samples (approximately 5 g of dry weight) were homogenized into a fine paste. Methanol (analytical grade, ≥99.9%; RCI Labscan Ltd., Bangkok, Thailand) was used as the extraction solvent at a sample-to-solvent ratio of 1:2 (*w*/*v*). The mixtures were shaken on an orbital shaker at 250 rpm at 25 °C for 12 h (overnight). A single extraction cycle was performed, after which the mixtures were centrifuged at 3500 rpm for 10 min. The supernatants were collected and concentrated using a rotary evaporator at 40 °C under reduced pressure until the solvent was completely removed. The resulting crude extract was weighed to calculate the extraction yield (%), using the following Equation (1):

Extraction yield (%) = (Weight of dry crude extract/Weight of dry pollen) × 100(1)

The dried crude extracts were then redissolved in 10 mL of methanol and stored at 4 °C until further analysis [15].

### 2.5. Antioxidant Activity and Total Polyphenol Determination

The methanolic bee pollen extracts (from *Mimosa* spp. and *Xyris* spp.) were redissolved in methanol and serially diluted to obtain working concentrations ranging from 0.078125 to 10 mg/mL for the antioxidant assays. All absorbance measurements were performed using a SPECTROstar Nano microplate reader (BMG LABTECH GmbH, Ortenberg, Germany). All assays were performed in triplicate (*n* = 3), and results were expressed on a dry pollen weight basis.

#### 2.5.1. DPPH Radical Scavenging Activity

The free radical scavenging activity of the methanolic bee pollen extracts was determined using the DPPH (2,2-diphenyl-1-picrylhydrazyl) assay, following the previously method [16] with slight modifications. Ascorbic acid was used as the reference standard, with serial dilutions ranging from 0 to 100 µg/mL prepared for calibration. Briefly, the DPPH stock solution was prepared by dissolving 24 mg of DPPH in 100 mL of methanol (100%), and the working solution was diluted with methanol to obtain an absorbance of 1.1 ± 0.02 at 515 nm. Then, 100 µL of each sample extract or standard was pipetted into a 96-well plate, followed by the addition of 100 µL of working DPPH solution. The plate was incubated in the dark at room temperature for 30 min, and absorbance was measured at 515 nm using a microplate reader. The percentage of inhibition was calculated using Equation (2):

DPPH inhibition (%) = [(A_control − A_sample)/A_control] × 100(2)

where A_control is the absorbance of the blank and A_sample is the absorbance of the sample. The IC_50_ value, representing the concentration of extract required to scavenge 50% of DPPH radicals, was calculated from the dose–response curve.

#### 2.5.2. ABTS Radical Scavenging Activity

The ABTS radical scavenging activity was measured following the previous method [17,18] with modifications. The ABTS stock solution was prepared by dissolving 19.5 mg of ABTS and 3.3 mg of potassium persulfate (K_2_S_2_O_8_) in 7 mL of distilled water and incubating in the dark at room temperature for 12–16 h. Trolox was used as the standard, with working concentrations ranging from 0 to 1000 µM. The ABTS working solution was diluted with 80% ethanol to obtain an absorbance of 0.70 ± 0.02 at 734 nm. For the assay, 30 µL of sample extract or Trolox standard was added to a 96-well plate, followed by 170 µL of the ABTS working solution. The plate was incubated in the dark for 10 min, and absorbance was measured at 734 nm. The percentage of inhibition was calculated using Equation (3):

ABTS inhibition (%) = [(A_control − A_sample)/A_control] × 100(3)

The IC_50_ value was determined from the linear regression of the dose–response curve. Where A_control is the absorbance of the ABTS working solution without sample and A_sample is the absorbance of the reaction mixture containing the sample extract or standard.

#### 2.5.3. Ferric Reducing Antioxidant Power (FRAP) Assay

The FRAP assay was adapted from a previously published method [19] to evaluate the electron-donating capacity of the bee pollen extracts. The FRAP reagent was freshly prepared by mixing acetate buffer (300 mM, pH 3.6), 10 mM TPTZ in 40 mM HCl, and 20 mM FeCl_3_·6H_2_O in a 10:1:1 ratio. Ascorbic acid standards (0–1000 µg/mL) were prepared for calibration. For analysis, 10 µL of standard or sample extract was added to a 96-well plate, followed by 190 µL of FRAP reagent. The plate was incubated in the dark for 30 min at room temperature, and absorbance was measured at 593 nm. FRAP values were expressed as milligrams of ascorbic acid equivalents per 100 g of dry pollen (mg AAE/100 g dry pollen), calculated using Equation (4):

FRAP (mg AAE/100 g dry pollen) = (C_AA × V × DF × 100)/W(4)

where C_AA is the ascorbic acid concentration derived from the standard curve (mg/mL), V is the volume of the extract solution (mL), DF is the dilution factor, and W is the dry weight of pollen (g).

#### 2.5.4. Total Phenolic Content (TPC)

The total phenolic content of the methanolic bee pollen extracts was determined using the Folin–Ciocalteu method, adapted from a published method [18,20,21]. Gallic acid (0–200 µg/mL) was used as the reference standard. Briefly, 12.5 µL of the sample extract was mixed with 13 µL of deionized water, followed by the addition of 13 µL of Folin–Ciocalteu reagent. The mixture was incubated at room temperature for 6 min, after which 125 µL of 7% sodium carbonate solution and 100 µL of distilled water were added. The absorbance was measured at 760 nm using a microplate reader after 30 min of incubation in the dark. The TPC was calculated using Equation (5) and expressed as milligrams of gallic acid equivalents per gram of dry pollen (mg GAE/g dry pollen):

TPC (mg GAE/g dry pollen) = (C_GA × V × DF)/W(5)

where C_GA is the concentration of gallic acid from the standard curve (mg/mL), V is the volume of extract solution (mL), DF is the dilution factor, and W is the dry weight of pollen (g).

#### 2.5.5. Total Flavonoid Content (TFC)

The total flavonoid content was measured by a colorimetric assay based on the previously method [18,22]. Quercetin was used as the reference standard, with calibration concentrations ranging from 0 to 100 µg/mL. The methanolic bee pollen extracts were prepared at 1 mg/mL in methanol. Briefly, 25 µL of the sample extract was added to a 96-well plate, followed by 8 µL of 7% sodium nitrite solution and 13 µL of distilled water. The mixture was allowed to stand at room temperature for 5 min. Then, 15 µL of 10% aluminum chloride solution was added, mixed thoroughly, and incubated at room temperature for an additional 5 min. Subsequently, 50 µL of 1 M sodium hydroxide solution and 28 µL of distilled water were added, and the mixture was incubated at room temperature for 5 min. The absorbance was measured at 510 nm using a microplate reader, with distilled water as the blank. The TFC was calculated using Equation (6) and expressed as milligrams of quercetin equivalents per gram of dry pollen (mg QE/g dry pollen):

TFC (mg QE/g dry pollen) = (C_QE × V × DF)/W(6)

where C_QE is the concentration of quercetin from the standard curve (mg/mL), V is the volume of extract solution (mL), DF is the dilution factor, and W is the dry weight of pollen (g).

### 2.6. Biological Activity of Bee Pollen Extract

#### 2.6.1. Erythrocyte Oxidative Hemolysis Protection Assay

The anti-hemolytic effects of honeybee pollen extracts (125, 250, and 500 µg/mL) were evaluated on oxidized human erythrocytes [22]. Hemolysis of erythrocytes was induced by the thermal decomposition of AAPH in peroxyl radicals and was read at 540 nm AMR-100 microplate reader (Allsheng Instruments Co., Ltd., Hangzhou, China). Vitamin C was used as the standard. Results were expressed as a percentage of hemolysis compared to the control, referring to AAPH-treated erythrocytes. All experiments were performed in triplicate (*n* = 3).

#### 2.6.2. In Vitro Anti-Inflammatory Activity

Since human red blood cell (HRBC) membranes are similar to these lysosomal membrane components, the prevention of hypotonicity-induced HRBC membrane lysis was taken as a measure in estimating the anti-inflammatory property of pollen from different plant origins. Thus, the human red blood cell membrane stabilization (HRBC method) [18,23] was used as a method for estimating the anti-inflammatory property. All experiments were performed in triplicate (*n* = 3).

### 2.7. Phytochemical Compounds Analysis by LC-QToF-MS

The phytochemical profile of the methanolic bee pollen extracts was analyzed following a previously published method [16,18,21] with slight modifications. The crude extracts were redissolved in LC-MS-grade methanol at 1 mg/mL, cleaned up using a dispersive C18 SPE cartridge, and filtered through a 0.22 µm PTFE syringe filter. An aliquot of 1 µL was injected into an Agilent 1290 Infinity II UHPLC coupled to an Agilent 6546 LC-QToF-MS (Agilent Technologies, Santa Clara, CA, USA). Separation was performed on a ZORBAX Eclipse Plus C18 column (2.1 × 150 mm, 1.8 µm; Agilent Technologies, Santa Clara, CA, USA) at 35 °C with a flow rate of 0.2 mL/min. The mobile phase consisted of (A) 0.1% formic acid in water and (B) acetonitrile, with a gradient from 5% to 95% B over 30 min, followed by a 5 min re-equilibration. Detection was performed using electrospray ionization (ESI) in positive mode (capillary voltage, 4500 V; drying gas, 7 L/min N_2_ at 300 °C; nebulizer, 20 psi) in full-scan mode over *m*/*z* 50–1000. Data were processed using Agilent MassHunter Qualitative Analysis software version 10.0 (Agilent Technologies, Santa Clara, CA, USA) with the Find-by-Formula algorithm, and accurate masses were matched against the Agilent METLIN Metabolomics Database (METLIN library). Only features with a database matching score ≥ 90% and mass error ≤ 5 ppm were retained as putative formula/database matches (Tables S1 and S2). Because authentic standards and MS/MS fragmentation spectra were not used in this study, these compounds were not assigned as Metabolomics Standards Initiative (MSI) Level 2 identifications. They should therefore be interpreted as tentative candidates based on accurate mass and database matching.

### 2.8. Statistical Analysis

All experiments were performed in triplicate *(n* = 3), and results are expressed as mean ± standard deviation (SD). Statistical analyses were performed using IBM SPSS Statistics software version 22.0 (IBM Corp., Armonk, NY, USA). Before comparative analysis, the normality of data distribution was assessed using the Shapiro–Wilk and Kolmogorov–Smirnov tests. For comparisons between the two pollen types (*Mimosa* spp. vs. *Xyris* spp.), data showing a normal distribution were analyzed using the independent-samples *t*-test, whereas data that did not meet the assumption of normality were analyzed using the non-parametric Mann–Whitney U test. For the erythrocyte oxidative hemolysis protection and HRBC membrane stabilization assays, differences among the three concentrations (125, 250 and 500 µg/mL) within each pollen group were analyzed using one-way analysis of variance (ANOVA) followed by Tukey’s and Duncan’s post-hoc tests, and differences between the two pollen types at each concentration were analyzed using the independent-samples *t*-test or Mann–Whitney U test, depending on the outcome of the normality test. Differences were considered statistically significant at *p* < 0.05.

## 3. Results and Discussion

### 3.1. Botanical Identification and Morphological Characteristics of Bee Pollen

The botanical origin of the bee pollen samples was identified based on macroscopic characteristics and SEM analysis (Table 1). The two plant taxa, *Mimosa* spp. and *Xyris* spp., exhibited clearly different floral and pollen morphologies. *Mimosa* spp. flowers were characterized by spherical pink inflorescences with numerous fine stamens, whereas *Xyris* spp. exhibited small yellow flowers with a more compact floral structure. The bee pollen pellets also differed in physical characteristics: *Mimosa* spp. appeared as compact, granular pellets of relatively uniform size with yellowish-brown coloration, while *Xyris* spp. pellets showed differences in granule size and surface texture, consistent with their distinct botanical origin.

SEM analysis further confirmed these differences. At 2000× magnification, both pollen types exhibited distinct shapes and aggregation patterns. At higher magnifications (5000× and 10,000×), *Mimosa* pollen grains were rounded to slightly oval with a relatively smooth to finely textured surface, whereas *Xyris* pollen grains were more elongated with a clearly defined exine structure and a more pronounced surface ornamentation. These morphological observations are consistent with previous palynological descriptions of *Mimosa pudica* and *Xyris complanata* pollens from Brazilian and Thai sources [9,10]. The clear morphological distinction between the two pollen types provides the structural basis for downstream monofloral classification and demonstrates the suitability of SEM for detailed pollen characterization in studies where formal palynological enumeration is not performed [9]. The monofloral classification adopted in this study was therefore based on macroscopic color sorting and SEM-based morphological comparison against a pre-established pollen reference collection, using the threshold of >90% pollen frequency from a single plant taxon, as proposed in similar studies on monofloral Thai bee pollen [10].

### 3.2. Proximate Composition of Mimosa spp. and Xyris spp. Bee Pollen

The proximate composition of *Mimosa* spp. and *Xyris* spp. bee pollen showed significant differences in most nutritional parameters (Table 2). *Mimosa* spp. pollen had significantly higher crude protein (29.54 ± 0.44%) than *Xyris* spp. (18.36 ± 0.17%, *p* = 0.007), as well as higher ash (2.98 vs. 1.78%) and moisture (11.43 vs. 6.59%) contents. In contrast, *Xyris* spp. pollen showed significantly higher dry matter (93.41 vs. 88.57%), crude fat (5.02 vs. 1.25%), nitrogen-free extract (NFE; 68.72 vs. 55.06%), and gross energy (4824 vs. 4651 cal/g) values (*p* < 0.05). Crude fiber was not detected in either pollen sample. In this context, crude fiber refers mainly to acid- and alkali-insoluble lignocellulosic residues. The N.D. result therefore indicates that crude fiber was below the detection limit of the analytical method, possibly due to the low proportion of resistant fibrous residues or relatively thin pollen wall structures in these samples.

The crude protein content of *Mimosa* spp. (29.54%) falls within the upper range reported for monofloral bee pollens worldwide. Monofloral *Mimosa pudica* bee pollen from northeastern Brazil has been reported to contain approximately 21% protein [10], while Brazilian bee pollens dominated by *M. pudica* contained 16–27% protein [11]. The value obtained in our study also exceeds the typical average of ~23% reported in a comprehensive review of monofloral bee pollens worldwide [7], suggesting that Thai *Mimosa* spp. pollen is a particularly protein-rich resource. This is consistent with the typical Fabaceae pollen profile [13] and supports its ecological role as a high-protein resource for *Apis mellifera* during the dry-cool season in northern Thailand, when alternative high-protein floral sources are scarce.

In contrast, the lower protein and higher carbohydrate and lipid contents of *Xyris* spp. resemble the profile of pollens from grass-like and lowland herbaceous floral sources [24]. The higher NFE (68.72%) and crude fat (5.02%) values account for the greater gross energy density observed in *Xyris* spp. (4824 cal/g), suggesting that this pollen could serve primarily as an energy resource rather than a protein source for honey bee colonies. The absence of crude fiber in both samples is consistent with bee pollen of non-fibrous plant origin [6] and indicates good digestibility—a positive attribute for both colony nutrition and downstream functional applications. Together, these compositional differences clearly demonstrate that botanical origin shapes the nutritional profile of bee pollen, in agreement with previous reports comparing monofloral pollens of different floral sources [6,7,12,25].

### 3.3. Extraction Yield of Methanolic Bee Pollen Extracts

The methanolic extraction yield of *Xyris* spp. pollen (44.26%) was substantially higher than that of *Mimosa* spp. (34.63%; Table 3). This 1.3-fold difference is consistent with the higher non-structural carbohydrate (NFE) and lipid fractions in *Xyris* spp. (Section 3.2), which are efficiently solubilized by methanol [7]. The lower yield observed in *Mimosa* spp. likely reflects a higher proportion of insoluble or protein-bound material—consistent with its higher crude protein content—which is less extractable with methanol as a single solvent. Similar variations in extraction yield among monofloral bee pollens of different botanical origins have been documented previously, with values typically ranging between 20% and 50% depending on the floral source and the polarity of the extraction solvent [7,12].

Importantly, a higher gravimetric extraction yield does not necessarily reflect a higher content of bioactive secondary metabolites, as the crude extract is a mixture of bulk soluble compounds (sugars, lipids, free amino acids) and bioactive secondary metabolites (phenolics, flavonoids, alkaloids). This is clearly supported by our subsequent findings showing that *Mimosa* spp., despite its lower extraction yield, exhibited substantially higher phenolic content and antioxidant activity than *Xyris* spp. A similar dissociation between gross extract yield and bioactive content has been reported for monofloral bee pollens from Algeria [12] and Brazil [11,25], where extracts with higher gravimetric yields did not necessarily exhibit the strongest bioactivity. This emphasizes the importance of evaluating functional bioactivity directly through targeted assays rather than relying on extraction yield as a proxy for functional potential—a point that is particularly relevant when selecting bee pollens for further pharmacological or nutritional evaluation.

### 3.4. Antioxidant Activity of Methanolic Bee Pollen Extracts

The antioxidant activity of the methanolic bee pollen extracts was assessed using three complementary assays (Table 4). *Mimosa* spp. exhibited significantly lower DPPH IC_50_ (0.46 ± 0.002 mg/mL) and ABTS IC_50_ (26.74 ± 1.80 mg/mL) values than *Xyris* spp. (0.93 ± 0.030 and 129.72 ± 12.75 mg/mL, respectively; *p* < 0.05), indicating a markedly stronger radical-scavenging capacity. The FRAP value of *Mimosa* spp. (120.56 ± 0.24 mg AAE/100 g dry pollen) was also nearly twice that of *Xyris* spp. (61.15 ± 0.80 mg AAE/100 g dry pollen; *p* < 0.001), reflecting its superior electron-donating capacity. Together, the three assays consistently identified *Mimosa* spp. as the more potent radical scavenger and reducer.

The DPPH IC_50_ value of *Mimosa* spp. (0.46 mg/mL) is comparable to that of monofloral *Mimosa pudica* bee pollen from northeastern Brazil [10] and to high-phenolic monofloral pollens from Algeria [12] and Morocco [26], placing Thai *Mimosa* spp. pollen among the more active bee pollens documented to date. The DPPH IC_50_ of *Xyris* spp. (0.93 mg/mL) is comparable to that of Thai *Xyris complanata* bee pollen reported previously [9], which linked the moderate radical-scavenging activity of Thai bee pollens to their spermidine derivative content. The same study also reported notable antioxidant activity in *Mimosa pudica* extracts [27], further supporting the observation that *Mimosa*-derived bee pollens consistently exhibit strong antioxidant capacity regardless of geographical origin.

The IC_50_ values obtained from the DPPH and ABTS assays differed substantially in our study (DPPH: 0.46–0.93 mg/mL vs. ABTS: 26.74–129.72 mg/mL). This is consistent with the well-documented mechanistic and physicochemical differences between the two radicals [28,29]. The DPPH radical is bulky and sterically hindered, reacting preferentially with phenolic compounds in methanolic media via a mixed HAT/SET mechanism, whereas the ABTS radical cation is smaller, more soluble in both aqueous and organic media, and reacts more rapidly with a wider range of antioxidants via the SPLET mechanism [28,29]. Consequently, an extract that efficiently scavenges DPPH at low concentrations may require markedly higher concentrations to reach 50% scavenging of ABTS, and inter-assay discrepancies of 5- to 100-fold have been frequently reported in plant and pollen extracts [30,31]. Despite this difference in absolute IC_50_ values, both assays consistently indicated that *Mimosa* spp. pollen possesses stronger radical-scavenging activity than *Xyris* spp., supporting the robustness of our conclusion. Similar findings were recently reported in a study on the antioxidant (DPPH, ABTS, FRAP, TPC, TFC) and anti-inflammatory activities of *Apis cerana* honey from Thailand using comparable methodologies [32]. Those results, which also showed considerable variation in IC_50_ values among samples of different botanical origins, support our observation that the bioactive potential of honeybee-derived products is strongly influenced by floral source and geographical origin.

The FRAP value of *Mimosa* spp. (120.56 mg AAE/100 g dry pollen) lies within the upper range reported for monofloral pollens (typically 30–150 mg AAE/100 g) [7], whereas the *Xyris* spp. value (61.15 mg AAE/100 g) lies near the lower end of this range. This pattern is consistent with the higher TPC and TFC observed in *Mimosa* spp. (Section 3.5), suggesting that the reducing capacity of the extracts is largely driven by their phenolic and flavonoid content, as also reported for monofloral bee pollens from Morocco [26] and Algeria [12].

### 3.5. Total Phenolic and Flavonoid Contents

The *Mimosa* spp. extract showed significantly higher TPC (17.07 ± 0.19 mg GAE/g dry pollen) and TFC (8.89 ± 0.59 mg QE/g dry pollen) than *Xyris* spp. (10.39 ± 0.27 mg GAE/g and 6.94 ± 0.22 mg QE/g, respectively; *p* < 0.05; Table 5). This represents a 1.6-fold higher TPC and a 1.3-fold higher TFC in *Mimosa* spp., indicating a substantially richer profile of phenolic and flavonoid secondary metabolites.

These TPC values are consistent with the range reported for monofloral bee pollens in the international literature: 5–25 mg GAE/g for European, Brazilian, and North African pollens [7,12,25], and 8–20 mg GAE/g for Thai bee pollens [9]. The TPC of *Mimosa* spp. (17.07 mg GAE/g) is comparable to that reported for monofloral *Mimosa pudica* bee pollen from Brazil [10], confirming that *Mimosa*-derived bee pollen is consistently among the more phenolic-rich pollens across geographical regions and apparently irrespective of climatic conditions. The TFC values obtained in this study (6.94–8.89 mg QE/g) are also within the range previously reported (1–15 mg QE/g) for monofloral bee pollens [7,26], with *Mimosa* spp. lying in the upper part of that range.

The 1.6-fold higher TPC and 1.3-fold higher TFC in *Mimosa* spp. correspond well with its superior DPPH, ABTS, and FRAP performance (Section 3.4), supporting the widely reported correlation between phenolic/flavonoid content and antioxidant activity in bee pollen [7,12,25]. Mechanistically, phenolic compounds and flavonoids act as electron donors and hydrogen-atom donors capable of neutralizing reactive oxygen species, and their structural diversity (hydroxyl group position, degree of glycosylation, and conjugation pattern) directly modulates their ability to scavenge different types of radicals [28,29]. The higher phenolic and flavonoid content of *Mimosa* spp. likely reflects the contribution of secondary metabolites characteristic of Fabaceae floral sources, including hydroxycinnamic acid amides and flavonol glycosides previously reported in *M. pudica* pollen [10]. The relationship between TPC/TFC and bioactivity is further summarized in Section 3.7.

### 3.6. Erythrocyte Oxidative Hemolysis Protection and HRBC Membrane Stabilization Activity

**Figure 3:**
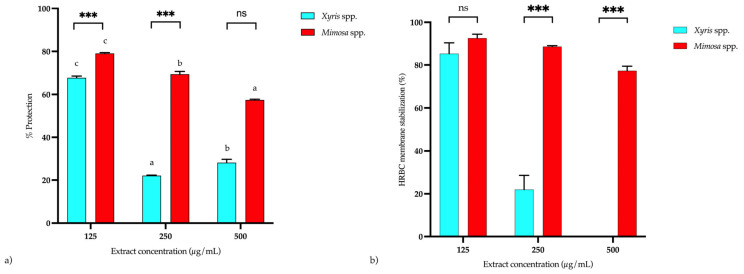
Biological activities of methanolic bee pollen extracts from *Xyris* spp. and *Mimosa* spp. at different extract concentrations; (**a**) erythrocyte oxidative hemolysis protection and (**b**) HRBC membrane stabilization activity. Values are expressed as mean ± SD (*n* = 3). Different lowercase letters above bars indicate significant differences among extract concentrations within the same plant origin and assay (*p* < 0.05); bars sharing the same letter are not significantly different. Asterisks indicate significant differences between plant origins at the same extract concentration: *** *p* < 0.001; ns, not significant.

Both extracts showed concentration-dependent effects in the oxidative hemolysis protection and HRBC membrane stabilization assays (Figure 3), but the patterns differed substantially between the two pollens. At 125 µg/mL, *Mimosa* spp. provided 79.03 ± 0.35% hemolysis protection and 92.61 ± 1.81% HRBC stabilization, compared to 67.73 ± 0.83% and 85.29 ± 5.16% for *Xyris* spp. *Mimosa* spp. exhibited a gradual concentration-dependent decline, retaining substantial activity at 500 µg/mL (57.05% hemolysis protection and 77.36% HRBC stabilization). In contrast, *Xyris* spp. showed a sharp drop at higher concentrations, with hemolysis protection decreasing to 22.00% (250 µg/mL) and 28.14% (500 µg/mL), and HRBC stabilization decreasing to 22.00% and 0.00%, respectively.

The membrane-protective activity of *Mimosa* spp. pollen is consistent with previous reports for phenolic-rich plant extracts on AAPH-induced erythrocyte hemolysis [22]. The HRBC membrane stabilization values of *Mimosa* spp. (77–92%) are comparable to those reported for ethanolic extracts of *Centella asiatica* [22] at similar concentrations, suggesting that *Mimosa* spp. bee pollen displays anti-inflammatory potential of a similar order of magnitude to these well-characterized medicinal plant extracts in this in vitro system. Mechanistically, phenolic compounds and flavonoids are known to stabilize erythrocyte membranes by intercalating into the phospholipid bilayer, scavenging peroxyl radicals generated by AAPH thermal decomposition, and preventing lipid peroxidation cascades [6]. The greater membrane-protective activity of *Mimosa* spp. across the tested concentration range is therefore consistent with its higher TPC and TFC reported in Section 3.5.

The non-monotonic dose–response of *Xyris* spp.—peaking at 125 µg/mL and decreasing sharply at higher concentrations—is consistent with the biphasic (hormetic) dose–response widely reported for plant phenolic and flavonoid extracts, in which compounds act as antioxidants at low concentrations but can switch to pro-oxidant behaviour at higher concentrations through polyphenol auto-oxidation, generation of hydrogen peroxide, and metal-catalyzed radical formation [33,34]. Such a pro-oxidant transition can compromise rather than protect erythrocyte membrane integrity. We acknowledge that the previous attribution of this effect to “cytotoxicity” was speculative, as no direct cytotoxicity assays (e.g., LDH leakage or MTT) or pro-oxidant indicator assays were performed in the present study. The observed decrease at higher concentrations is therefore more accurately described as a concentration-dependent loss of protective activity, possibly reflecting a hormetic or pro-oxidant transition. Confirmation of this mechanism will require dedicated cytotoxicity, pro-oxidant, and sub-haemolytic membrane-integrity assays in future studies.

Particularly, *Xyris* spp. extract showed a non-monotonic dose–response in both the hemolysis protection and HRBC membrane stabilization assays, with maximum activity at 125 µg/mL and a sharp decrease at higher concentrations. This pattern is consistent with the biphasic (hormetic) dose–response widely reported for plant phenolic and flavonoid extracts, in which compounds act as antioxidants at low concentrations but switch to pro-oxidant behaviour at higher concentrations through polyphenol auto-oxidation [33,34]. We acknowledge that our earlier attribution to “cytotoxicity” is speculative, as no direct cytotoxicity assays (e.g., LDH, MTT) were performed. The decrease at higher concentrations is therefore more accurately described as a concentration-dependent loss of protective activity, possibly reflecting a hormetic or pro-oxidant transition; confirmation will require dedicated cytotoxicity and pro-oxidant assays in future studies.

### 3.7. Descriptive Comparison of Phytochemical Content, Antioxidant Activity, and Biological Activity

Table 6 provides a descriptive comparison of the phytochemical content, antioxidant activity, and biological activity of the *Mimosa* spp. and *Xyris* spp. bee pollen extracts. Because the comparisons were based on condition-level mean values from only two botanical origins rather than multiple independent biological samples, the results are summarized qualitatively rather than through correlation analysis or statistical inference, in order to avoid overinterpreting the observed relationships.

As summarized in Table 6, the *Mimosa* spp. extract showed higher total phenolic content (TPC) and total flavonoid content (TFC) than the *Xyris* spp. extract. This was consistent with its stronger antioxidant activity, reflected by lower DPPH and ABTS IC_50_ values and higher FRAP activity. The *Mimosa* spp. extract also exhibited greater erythrocyte oxidative hemolysis protection and HRBC membrane stabilization across the tested concentrations, indicating stronger membrane-protective potential under the present experimental conditions. In contrast, the *Xyris* spp. extract showed lower TPC and TFC, together with weaker radical-scavenging, reducing, and membrane-protective activities.

Taken together, these descriptive trends suggest that the higher phenolic and flavonoid contents of *Mimosa* spp. may be associated with its stronger antioxidant and membrane-protective activities, consistent with the electron- and hydrogen-donating mechanisms widely reported for polyphenols [28,29] and with similar relationships described for bee pollen and other bee products [7,12,25,32]. However, this interpretation is descriptive only and should not be regarded as evidence of statistically robust correlations or causal relationships. Further studies using a larger number of independent bee pollen samples are needed to confirm these associations and to clarify the contribution of specific phytochemical constituents to the observed bioactivities.

### 3.8. Putative Phytochemical Annotation by LC-QToF-MS

The LC-QToF-MS analysis was performed to provide a preliminary phytochemical profile of the methanolic bee pollen extracts from *Xyris* spp. and *Mimosa* spp. The top ten putatively annotated compounds for each extract are presented in Tables S1 and S2. Several plant- and lipid-related compounds were annotated in the extracts, including farnesyl acetone, abscisic alcohol 11-glucoside, gingerenone B, eudesmin, kanzonol P, phytosphingosine, palmitic amide, and stearamide. These compounds are broadly related to plant secondary metabolism, lipid constituents, or phenolic/lignan-type metabolites, which may partly support the antioxidant and membrane-protective activities observed in the present study [1,2,3,4,9,10,12].

The presence of phenolic- and lignan-related annotations, such as gingerenone B, eudesmin, and kanzonol P, is consistent with the higher TPC, TFC, and antioxidant activity observed in *Mimosa* spp. extract. In addition, lipid-related compounds such as phytosphingosine, palmitic amide, and stearamide may contribute to the complex bioactive profile of bee pollen, since pollen contains both polar phenolic constituents and lipid-soluble metabolites [1,2,3,4]. These findings suggest that the biological activity of the extracts is likely derived from multiple classes of compounds rather than from a single phytochemical group.

However, all LC-QToF-MS results should be interpreted with caution. The compound assignments were based on accurate mass and database matching only, with matching scores ≥ 90% and mass error ≤ 5 ppm. No authentic standards or MS/MS fragmentation spectra were available for confirmation; therefore, these results correspond to putative annotations rather than confirmed compound identifications. Some drug-like or synthetic compound matches in the database are likely false-positive or isobaric matches and should not be interpreted as natural bee pollen constituents unless confirmed by authentic standards, MS/MS fragmentation, and appropriate blank controls. This cautious interpretation is important because database-based LC-MS annotation alone is insufficient for definitive structural identification.

Overall, the LC-QToF-MS results provide preliminary chemical evidence supporting the difference in bioactivity between *Xyris* spp. and *Mimosa* spp. bee pollen extracts. Nevertheless, future targeted metabolomic analysis, compound purification, MS/MS confirmation, and quantification are required to verify the annotated compounds and clarify their contribution to the antioxidant and biological activities of these bee pollens.

### 3.9. Limitations

Despite the encouraging results, this study has several limitations. First, the two pollen types were collected from different provinces: *Mimosa* spp. from Chiang Mai, northern Thailand, and *Xyris* spp. from Nong Bua Lam Phu, northeastern Thailand. These locations differ in climate, altitude, soil type, and surrounding vegetation. Therefore, the observed differences in bee pollen composition and bioactivity cannot be attributed to botanical origin alone, as geographical, climatic, edaphic, seasonal, and beekeeping factors may also influence pollen chemistry and biological properties [6,7,35]. Co-located sampling was not feasible because *Mimosa* spp. and *Xyris* spp. occupy non-overlapping habitats in Thailand. To partially reduce this limitation, both samples were collected during the same season, October–December 2023, using the same pollen-trap protocol on *Apis mellifera* colonies.

Second, the findings are limited by the small number of botanical sources examined. This study compared only two bee pollen types, *Xyris* spp. and *Mimosa* spp.; therefore, broader sampling from additional floral sources, apiaries, locations, and seasons is needed to better understand the variability of bee pollen composition and bioactivity. In addition, the biological activities were evaluated only using in vitro assays; therefore, the efficacy and safety of these pollen extracts under in vivo or real-world conditions remain unknown. Toxicity assessment should also be performed before considering future food, nutraceutical, or health-related applications.

Third, although LC-QToF-MS analysis putatively annotated several compounds, the study did not confirm or quantify individual compounds using authentic standards or MS/MS fragmentation. Therefore, the contribution of specific phytochemicals to antioxidant and membrane-protective activities remains unclear. Future studies should include targeted metabolomic analysis, compound confirmation, and quantitative determination of major bioactive constituents.

Finally, the monofloral classification of the bee pollen samples was based on macroscopic sorting and SEM-based morphological comparison with a reference collection. Formal palynological enumeration was not performed, such as counting at least 500 pollen grains across multiple microscopic fields to quantify the percentage of the dominant pollen type and identify accompanying pollen types. Future studies should incorporate systematic palynological counting to formally confirm the monofloral nature of bee pollen samples.

## 4. Conclusions

In conclusion, this study demonstrates that botanical origin influences the nutritional composition, phytochemical profile, antioxidant activity, and membrane-protective effects of Thai bee pollen. *Mimosa* spp. showed higher crude protein, TPC, and TFC than *Xyris* spp., together with stronger DPPH, ABTS, FRAP, erythrocyte hemolysis protection, and HRBC membrane stabilization activities. In contrast, *Xyris* spp. had higher crude fat, nitrogen-free extract, gross energy, and extraction yield.

The correlation analysis suggests that phenolic and flavonoid contents may contribute to the observed antioxidant and biological activities. LC-QToF-MS analysis provided preliminary phytochemical annotation of several plant- and lipid-related compounds; however, these results remain putative and require confirmation by MS/MS fragmentation, authentic standards, and quantitative analysis. Therefore, while *Mimosa* spp. bee pollen showed greater in vitro bioactivity under the tested conditions, further multi-site sampling, palynological confirmation, toxicity assessment, and in vivo studies are required before potential food or health-related applications can be considered.

## Figures and Tables

**Table 1:** Plant Origin and Bee Pollen Identification and Visual Documentation of Bee Pollen Samples.

	Plant Origin	
Scientific Name	*Mimosa* spp.	*Xyris* spp.
Character of bee pollen		
SEM 2000×	* 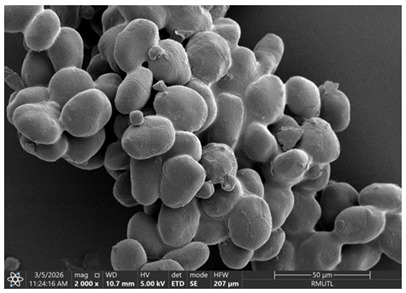 *	* 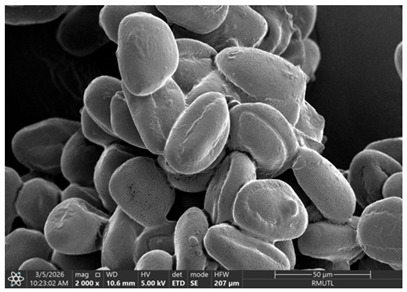 *
SEM 5000×	* 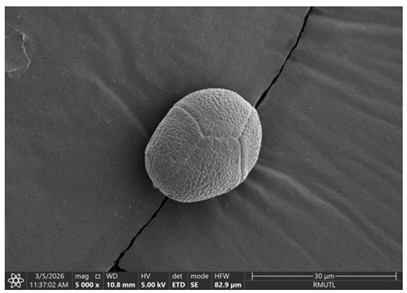 *	* 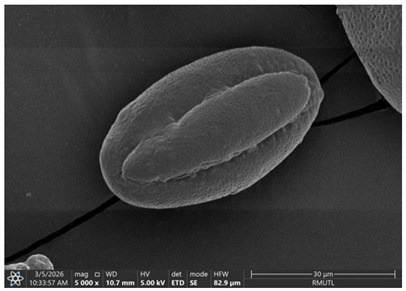 *
SEM 10,000×	* 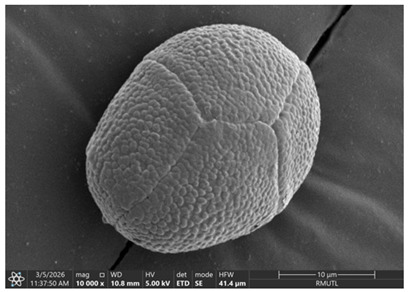 *	* 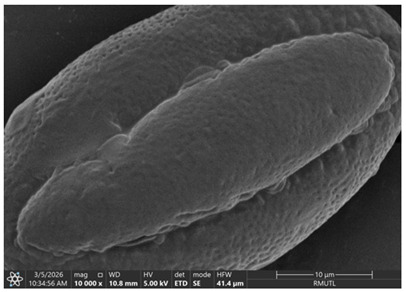 *

**Table 2:** *Mimosa* spp. and *Xyris* spp. Pollen Nutritional Composition.

Analysis	*Mimosa* spp.	*Xyris* spp.	*p*-Value
Dry matter (%)	88.57 ± 0.04 ^a^	93.41 ± 0.11 ^b^	0.004
Moisture (%)	11.43 ± 0.04 ^b^	6.59 ± 0.11 ^a^	0.004
Ash (%)	2.98 ± 0.01 ^b^	1.78 ± 0.00 ^a^	<0.001
Crude protein (%)	29.54 ± 0.44 ^b^	18.36 ± 0.17 ^a^	0.007
Crude fat (%)	1.25 ± 0.13 ^a^	5.02 ± 0.38 ^b^	0.029
Crude fiber (%)	N.D.	N.D.	N.D.
Nitrogen free extract (%)	55.06 ± 0.40 ^a^	68.72 ± 0.20 ^b^	0.003
Energy (cal/g)	4651.15 ± 16.33 ^a^	4824.25 ± 13.22 ^b^	0.008

Note: Values are expressed as mean ± SD (*n* = 3). Different lowercase superscript letters in the same row indicate significant differences between plant origins (*p* < 0.05). Statistical comparisons were performed using an independent-samples *t*-test or Mann–Whitney U test, depending on data normality. N.D., not detected; NFE, nitrogen-free extract. Crude fiber refers to acid- and alkali-insoluble fibrous residues, mainly lignocellulosic materials. The N.D. result indicates that crude fiber was below the detection limit of the analytical method used.

**Table 3:** Yield (%) of bee pollen methanolic crude extracted from different plant origins.

Plant Origin	Pollen Weight (g)	Crude Extracted (g)	Yield (%)
*Mimosa* spp.	5.08	1.76	34.63
*Xyris* spp.	5.06	2.24	44.26

**Table 4:** Bioactivity (antioxidant capacity) of methanolic bee pollen extracted from different plant origins.

Sample	DPPH		ABTS		FRAP	
	IC_50_ (mg/mL)	*p*-Value	IC_50_ (mg/mL)	*p*-Value	(mg AAE/100 g Dry Sample)	*p*-Value
*Mimosa* spp.	0.46 ± 0.002	0.046 *	26.74 ± 1.80	0.0044 *	120.56 ± 0.24	<0.001 *
*Xyris* spp.	0.93 ± 0.030		129.72 ± 12.75		61.15 ± 0.80	

Note: Values are expressed as mean ± SD (*n* = 3). An asterisk (*) indicates a significant difference between *Mimosa* spp. and *Xyris* spp. within the same assay (*p* < 0.05). DPPH data were analyzed using the Mann–Whitney U test. ABTS data were analyzed using Welch’s independent-samples *t*-test because of unequal variance between groups, whereas FRAP data were analyzed using the independent-samples *t*-test.

**Table 5:** The total phenolic and total flavonoid content of methanolic bee pollen extracted from different plant origins.

Sample	Total Phenolic Compound (mg GAE/g Dry Pollen)	*p*-Value	Total Flavonoid Content (mg QE/g Dry Pollen)	*p*-Value
*Mimosa* spp.	17.07 ± 0.19 ^b^	<0.001 *	8.89 ± 0.59 ^b^	0.006 *
*Xyris* spp.	10.39 ± 0.27 ^a^		6.94 ± 0.22 ^a^	

Note: Values are expressed as mean ± SD (*n* = 3). Different lowercase superscript letters in the same column indicate significant differences between plant origins by independent-samples t-test. * indicates a statistically significant difference (*p* < 0.05).

**Table 6:** Descriptive comparison of phytochemical content, antioxidant activity, and biological activity between *Mimosa* spp. and *Xyris* spp. bee pollen extracts.

Parameter	*Mimosa* spp.	*Xyris* spp.	Descriptive Interpretation
TPC	Higher	Lower	*Mimosa* spp. contained more phenolic compounds
TFC	Higher	Lower	*Mimosa* spp. contained more flavonoids
DPPH	Lower	Higher	Lower IC_50_ indicates stronger DPPH radical-scavenging activity in *Mimosa* spp.
ABTS	Lower	Higher	Lower IC_50_ indicates stronger ABTS radical-scavenging activity in *Mimosa* spp.
FRAP	Higher	Lower	*Mimosa* spp. showed higher reducing power
Erythrocyte oxidative hemolysis protection	Higher	Lower	*Mimosa* spp. showed stronger erythrocyte membrane-protective activity
HRBC membrane stabilization	Higher	Lower	*Mimosa* spp. showed stronger membrane stabilization activity

Note: This table summarizes descriptive trends based on the results presented in Table 4 and Table 5 and Figure 3. No statistical correlation analysis was performed because the dataset was based on condition-level mean values rather than multiple independent biological samples.

## Data Availability

The original contributions presented in this study are included in the article/Supplementary Materials. Further inquiries can be directed to the corresponding authors.

## Supplementary Materials

The following supporting information can be downloaded at: https://www.mdpi.com/article/10.3390/foods15111990/s1, Table S1: Top ten putatively annotated phytochemicals in bee pollen extracts from *Mimosa* spp. by LC-QToF-MS in positive ESI mode; Table S2: Top ten putatively annotated phytochemicals in bee pollen extracts from *Xyris* spp. by LC-QToF-MS in positive ESI mode. Figure S1. LC-QToF-MS chromatographic profile and extracted ion chromatograms of the top ten putatively annotated compounds in *Mimosa* spp. bee pollen extract ranked by database matching scores in positive ESI mode. Panel (a) shows the overall LC-QToF-MS chromatogram across the full retention time range, while panels (b–k) show the extracted ion chromatograms corresponding to the compounds listed in Table S1. Figure S2. LC-QToF-MS chromatographic profile and extracted ion chromatograms of the top ten putatively annotated compounds in *Xyris* spp. bee pollen extract ranked by database matching scores in positive ESI mode. Panel (a) shows the overall LC-QToF-MS chromatogram across the full retention time range, while panels (b–k) show the extracted ion chromatograms corresponding to the compounds listed in Table S2. References [36,37,38,39,40,41,42,43,44,45,46,47,48,49,50] are cited in Supplementary Materials.
