# Supplementary material for: Compositional and Biofunctional Properties of Xyris spp. and Mimosa spp. Bee Pollen from Thailand
Source: Foods. 2026 Jun 3;15(11):1990. doi: 10.3390/foods15111990 (PMC13257389; doi:10.3390/foods15111990)
Supplement: Supplementary file 1 [file foods-15-01990-s001.zip › foods-4296268-supplementary.pdf]

Table S1: Top ten putatively annotated phytochemicals in bee pollen extracts from *Mimosa* spp. by LC-QToF-MS in positive ESI mode.

No	Compound	Formula	RT (min)	Matching score (%)	Diff (DB, ppm)	m/z	Proposed adduct	Mass	Note
1	Farnesyl acetone	$C_{18}H_{30}O$	18.318	99.82	0.56	280.2636	$[M+NH_4]^+$	262.2298	Related to aromatic compounds and secondary metabolites from plants, farnesyl/hexahydrofarnesyl acetone compounds in plants have been reported to exhibit antioxidant, antibacterial, and anti-inflammatory activities [36].
2	Phytosphingosine	$C_{18}H_{39}NO_3$	1.864	99.79	0.24	318.3003	$[M+H]^+$	317.2931	Phytosphingosine has also been found to have interesting T-cell related ant-inflammatory properties in models of inflammatory bowel disease [37].
3	Palmitic amide	$C_{16}H_{33}NO$	20.297	99.77	0.61	256.2637	$[M+H]^+$	255.2564	Palmitamide is a fatty acid amide associated with anti-inflammatory, antioxidant, anti-allergic, or neuroprotective effects in some experimental systems [38].
4	Stearamide	$C_{18}H_{37}NO$	26.846	99.72	0.36	284.2949	$[M+H]^+$	283.2876	Is a fatty amide of stearic acid. It has a role as a metabolite. It is functionally related to an octadecanoic acid [39].
5	Gingerenone B	$C_{22}H_{26}O_6$	3.686	99.71	0.08	387.1805	$[M+H]^+$	386.1732	Gingerenone B belongs to the class of organic compounds known as linear diarylheptanoids. This could make gingerenone b a potential biomarker for the consumption of these foods [40].
6	Clausarinol	$C_{24}H_{30}NO_6$	5.488	99.62	0.57	415.2118	$[M+Na]^+$	414.2045	It is a natural substance from plants classified as linear pyranocoumarins / coumarin-related compound [41].

No	Compound	Formula	RT (min)	Matching score (%)	Diff (DB, ppm)	m/z	Proposed adduct	Mass	Note
7	Lobeline	$C_{22}H_{27}NO_2$	15.730	98.89	-0.28	338.2114	$[M+H]^+$	337.2042	Lobeline has multiple mechanisms of action, acting as a VMAT2 ligand, which stimulates dopamine release to a moderate extent when administered alone, but reduces the dopamine release caused by methamphetamine [42].
8	Kanzonol P	$C_{22}H_{24}O_5$	3.684	98.42	-0.36	369.1697	$[M+H]^+$	368.1624	Kanzonol P is a naturally occurring prenylated flavonoid belonging to the pterocarpan subclass [43].
9	Pectachol	$C_{26}H_{34}NO_6$	5.431	98.30	-0.17	460.2689	$[M+H]^+$	459.2618	Coumarin/sesquiterpene-coumarin derivative.<br>Natural product is terpenoid/sesquiterpene [44].
10	Palmitoleamide	$C_{16}H_{31}NO$	15.259	96.91	-0.14	254.2478	$[M+NH_4]^+$	253.2405	Palmitoleamide is a monounsaturated primary fatty acid amide synthesized from palmitoleic acid and ammonia [45].

Note: Compounds are reported as putatively annotated based on accurate mass and matching against the Agilent MassHunter METLIN Metabolomics Database (matching score ≥ 90%, mass error ≤ 5 ppm). No authentic standards or MS/MS fragmentation were used for confirmation. Therefore, these annotations correspond to Metabolomics Standards Initiative Level 2 of identification.

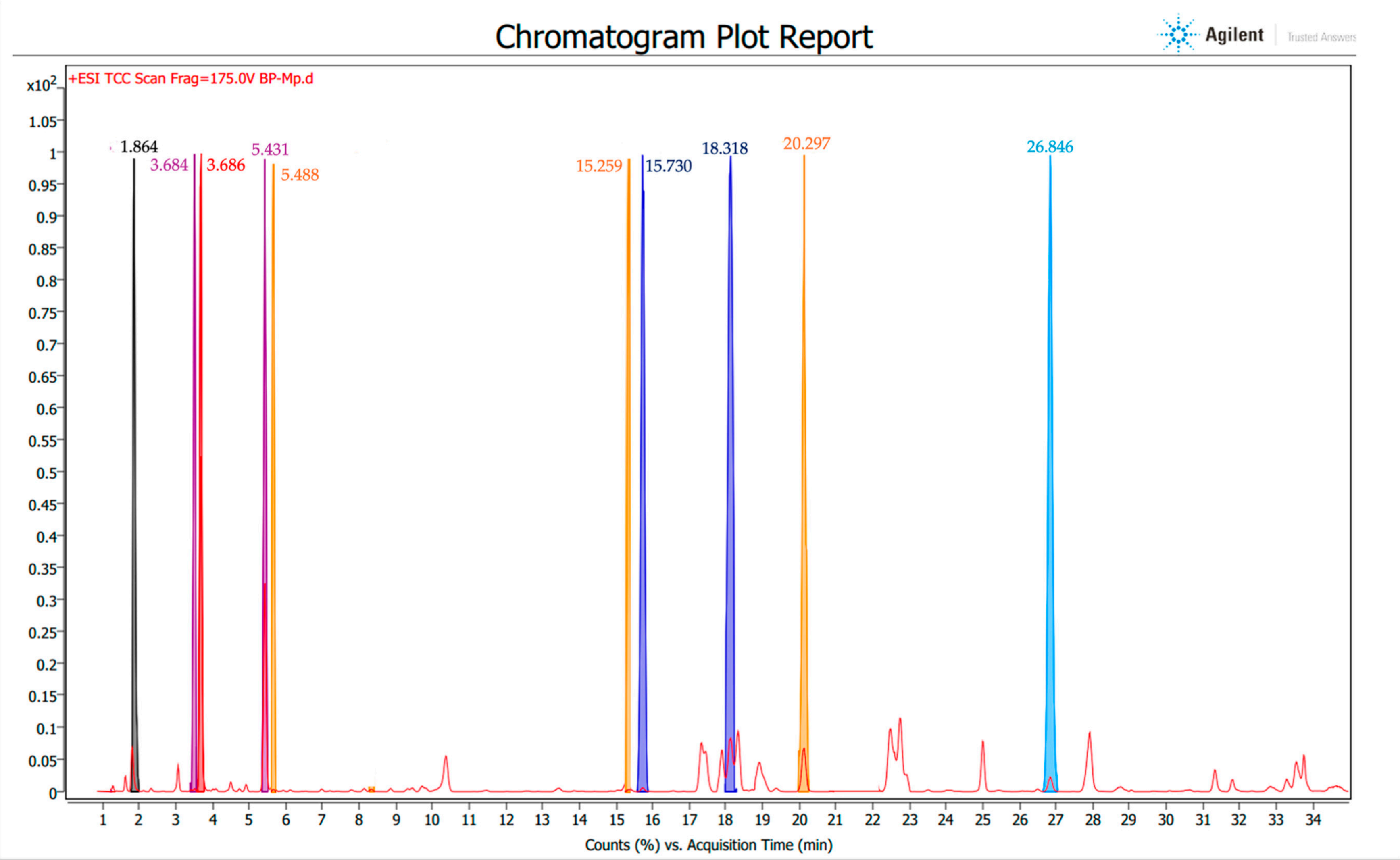
Chromatogram Plot Report
Agilent
Trusted Answers
+ESI TCC Scan Frag=175.0V BP-Mp.d
x10^2
1.864
3.684
3.686
5.431
5.488
15.259
15.730
18.318
20.297
26.846
Counts (%) vs. Acquisition Time (min)

a)

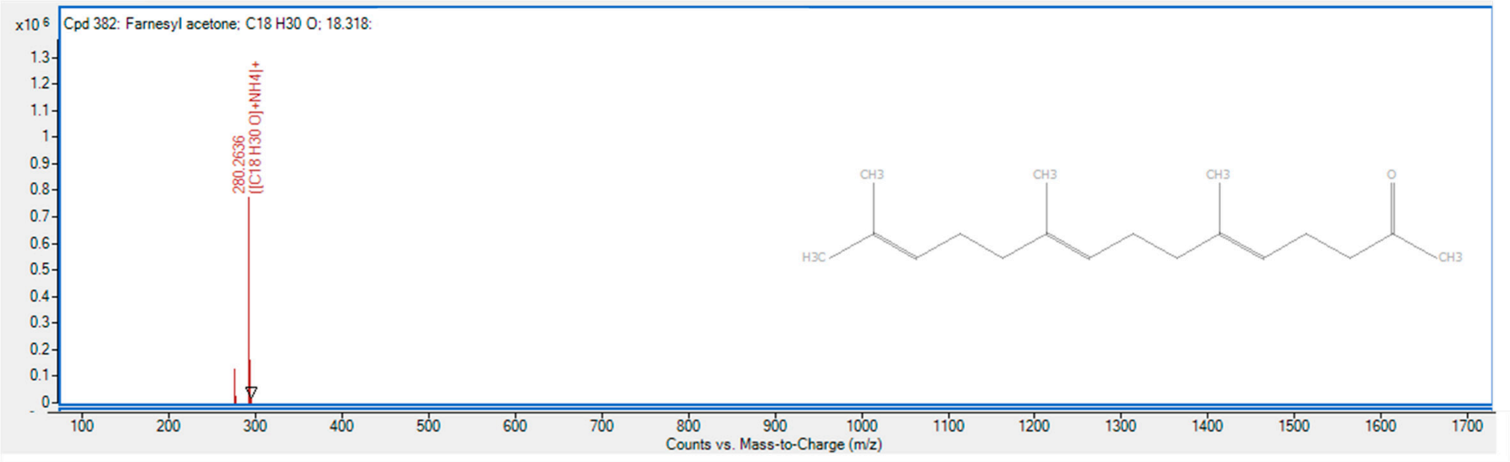

x10 6
Cpd 382: Farnesyl acetone; C18 H30 O; 18.318:
280.2636
([C18 H30 O]+NH4]+
CH3
CH3
CH3
O
H3C
CH3
Counts vs. Mass-to-Charge (m/z)

b)

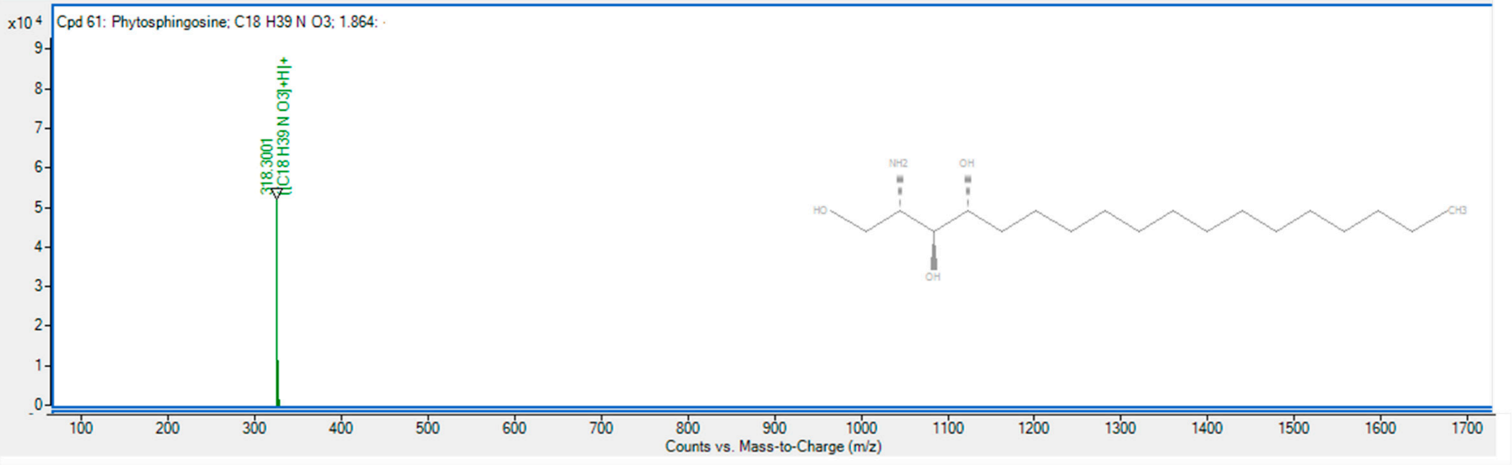

x10 4
Cpd 61: Phytosphingosine; C18 H39 N O3; 1.864:
318.3001
([C18 H39 N O3]+H]+
NH2
OH
HO
CH3
OH
Counts vs. Mass-to-Charge (m/z)

c)

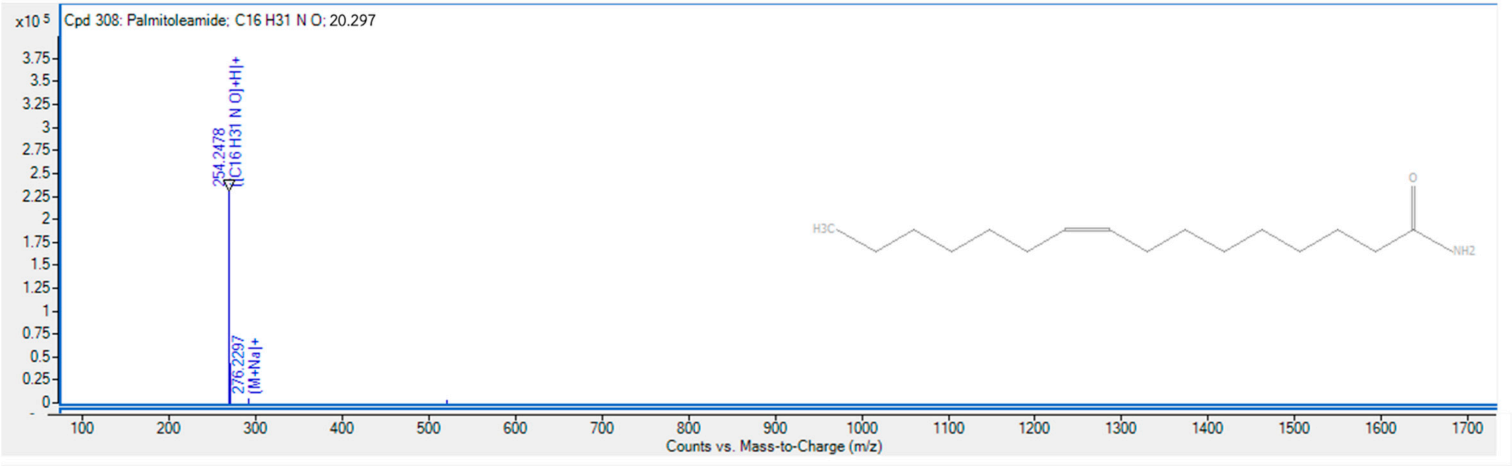

x10 5
Cpd 308: Palmitoleamide; C16 H31 N O; 20.297
254.2478
([C16 H31 N O]+H)+
276.2297
(M+Na)+
H3C
O
NH2
Counts vs. Mass-to-Charge (m/z)

d)

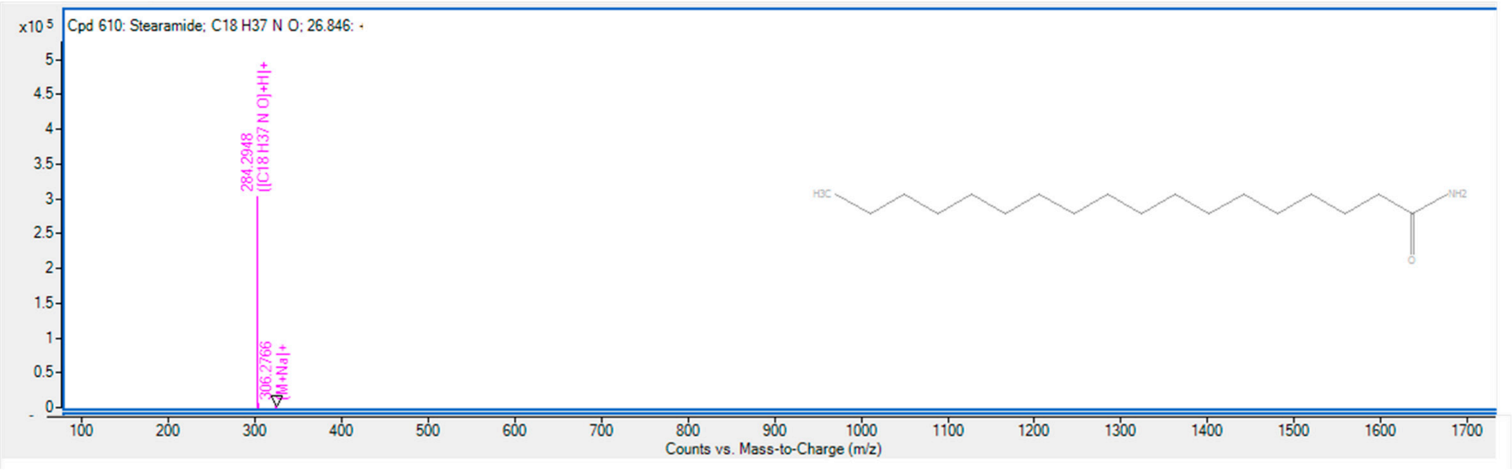

x10 5
Cpd 610: Stearamide; C18 H37 N O; 26.846: -
284.2948
([C18 H37 N O]+H)+
306.2766
(M+Na)+
H3C
NH2
O
Counts vs. Mass-to-Charge (m/z)

e)

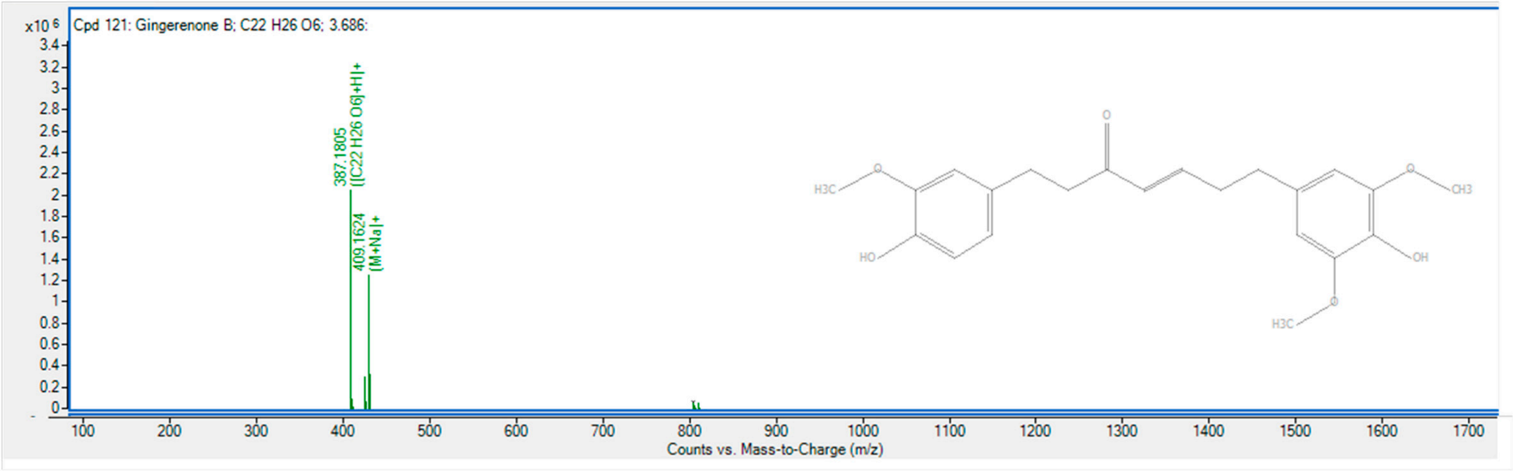
x10 6
Cpd 121: Gingerenone B; C22 H26 O6; 3.686:
387.1805
([C22 H26 O6]+H]+
409.1624
[M+Na]+
Counts vs. Mass-to-Charge (m/z)

f)

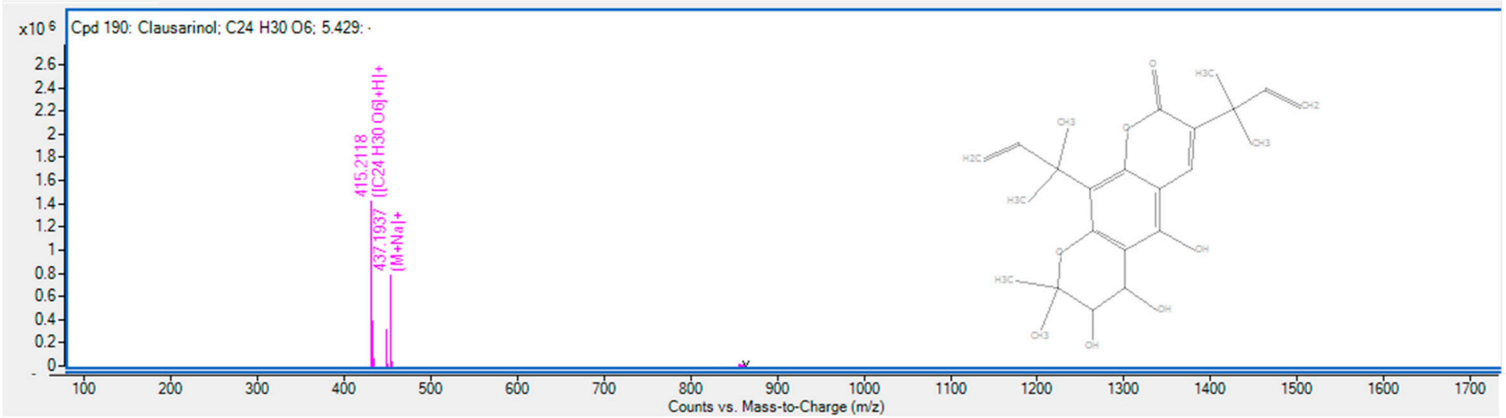
x10 6
Cpd 190: Clausarinol; C24 H30 O6; 5.429: ·
415.2118
([C24 H30 O6]+H]+
437.1937
[M+Na]+
Counts vs. Mass-to-Charge (m/z)

g)

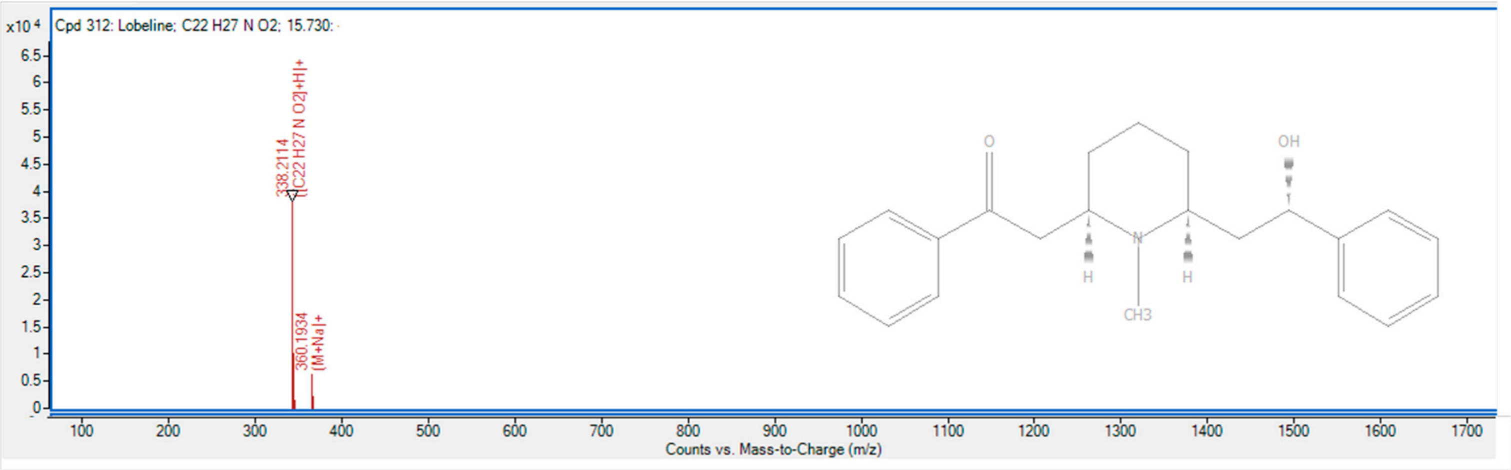
x10 4
Cpd 312: Lobeline; C22 H27 N O2; 15.730:
338.2114
([C22 H27 N O2]+H]+
360.1934
(M+Na]+
O
N
H
H
CH3
OH
Counts vs. Mass-to-Charge (m/z)

h)

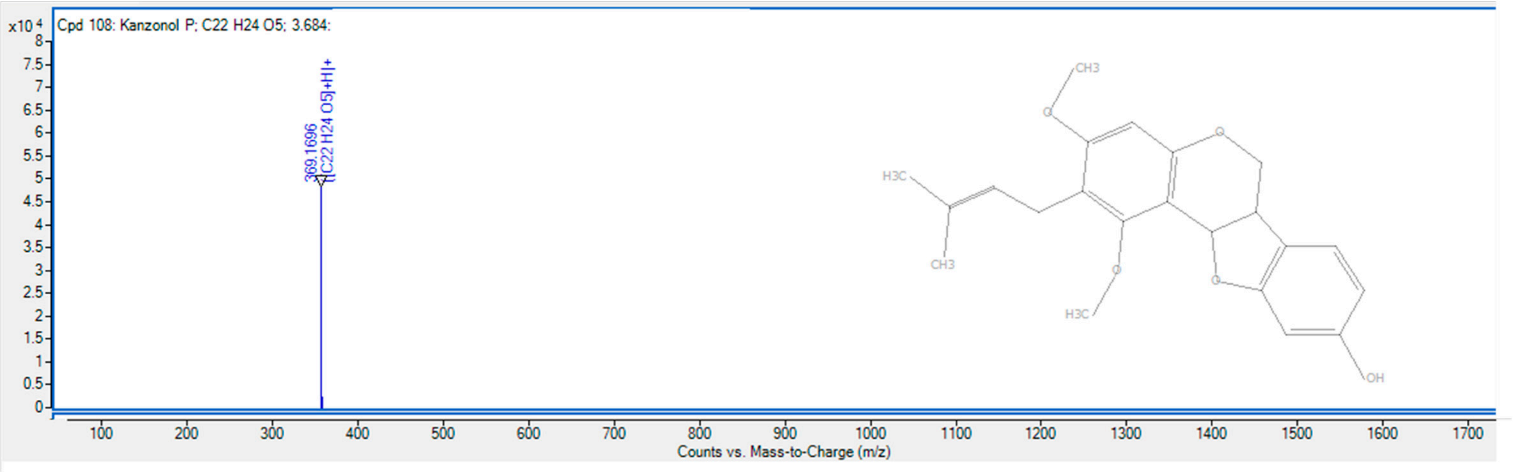
x10 4
Cpd 108: Kanzonol P; C22 H24 O5; 3.684:
369.1696
([C22 H24 O5]+H]+
CH3
O
H3C
CH3
O
H3C
O
O
OH
Counts vs. Mass-to-Charge (m/z)

i)

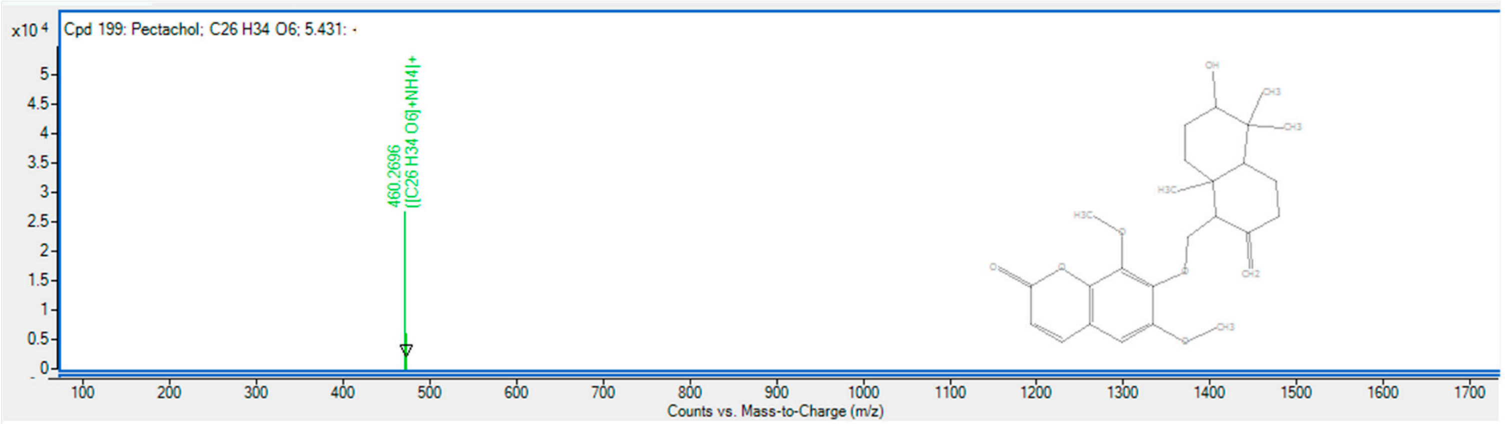

j)

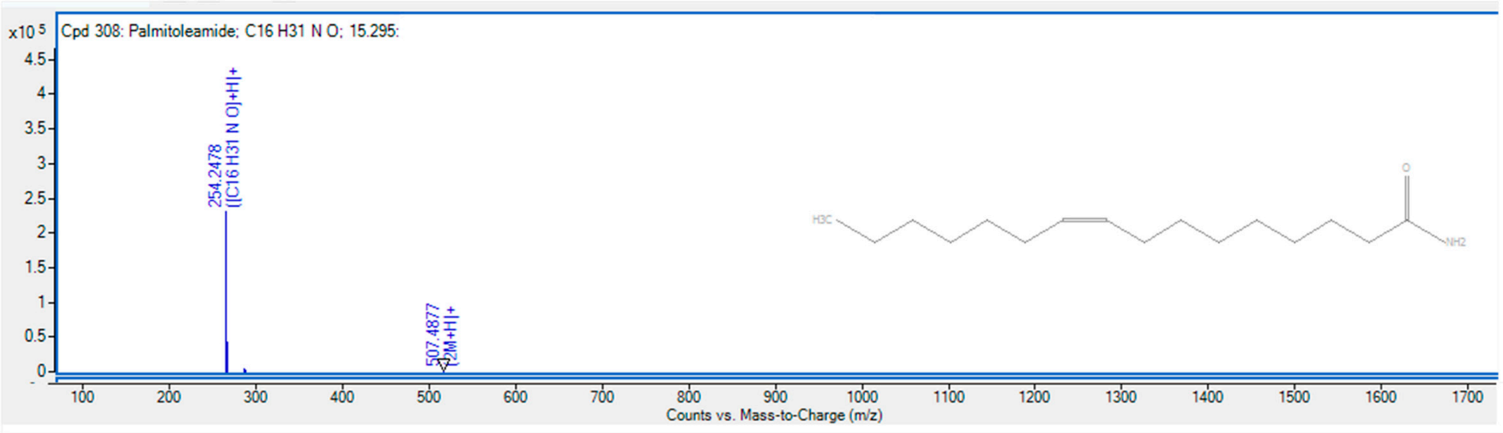

k)

Figure S1. LC-QToF-MS chromatographic profile and extracted ion chromatograms of the top ten putatively annotated compounds in *Mimosa* spp. bee pollen extract ranked by database matching scores in positive ESI mode. Panel (a) shows the overall LC-QToF-MS chromatogram across the full retention time range, while panels (b–k) show the extracted ion chromatograms corresponding to the compounds listed in Table S1.

Table S2: Top ten putatively annotated phytochemicals in bee pollen extracts from *Xyris* spp. by LC-QToF-MS in positive ESI mode.

No	Compound	Formula	RT (min)	Matching score (%)	Diff (DB, ppm)	m/z	Proposed adduct	Mass	Note
1	Farnesyl acetone	$C_{18}H_{30}O$	18.353	99.82	0.55	280.2636	$[M+NH_4]^+$	262.2298	Related to aromatic compounds and secondary metabolites from plants, farnesyl/hexahydrofarnesyl acetone compounds in plants have been reported to exhibit antioxidant, antibacterial, and anti-inflammatory activities [36].
2	Abscisic alcohol 11-glucoside	$C_{21}H_{32}O_8$	1.039	99.56	-0.12	430.2436	$[M+NH_4]^+$	412.2097	It is involved in the abscisic acid pathway and is an important phytohormone in plants. ABA plays a role in stress response, drought tolerance, and is involved in changes in flavonoid/lipid metabolism in plants [46].
3	Gingerenone B	$C_{22}H_{26}O_6$	3.698	99.27	0.65	387.1805	$[M+H]^+$	386.1732	Gingerenone B belongs to the class of organic compounds known as linear diarylheptanoids. This could make gingerenone b a potential biomarker for the consumption of these foods [40].
4	Falcarindiol 3-acetate	$C_{18}H_{30}O$	18.353	99.26	0.55	280.2636	$[M+NH_4]^+$	262.2298	Falcarindiol 3-acetate is a naturally occurring C17-polyacetylenic oxylipin predominantly [47].
5	Oleamide	$C_{18}H_{35}NO$	23.518	99.26	-0.03	282.2792	$[M+H]^+$	281.2719	Synthetic oleamide has many industrial applications, including as a lubricant [48].
6	Eudesmin	$C_{22}H_{26}O_6$	4.751	99.15	-0.27	409.162	$[M+Na]^+$	386.1728	Eudesmin have a broad range of biological activities including anti-bacterial, anti-inflammatory, anti-cancer, and anti-convulsant activity [49].
7	Lobeline	$C_{22}H_{27}NO_2$	15.756	98.83	-0.23	338.2114	$[M+H]^+$	337.2042	Lobeline has multiple mechanisms of action, acting as a VMAT2 ligand, which stimulates dopamine release to a moderate extent when administered alone, but

No	Compound	Formula	RT (min)	Matching score (%)	Diff (DB, ppm)	m/z	Proposed adduct	Mass	Note
									reduces the dopamine release caused by methamphetamine [42].
8	Kanzonol P	$C_{22}H_{24}O_5$	3.699	98.34	-0.45	369.1697	$[M+H]^+$	368.1624	Kanzonol P is a naturally occurring prenylated flavonoid belonging to the pterocarpan subclass [43].
9	Rutamarin	$C_{21}H_{24}O_5$	6.996	97.88	-0.03	379.1516	$[M+Na]^+$	356.1624	Rutamarin possessed the ability to kill cancer cells at a dose comparable to that of cisplatin [50].
10	Palmitoleamide	$C_{16}H_{31}NO$	15.259	95.40	-0.14	254.2478	$[M+NH_4]^+$	253.2405	Palmitoleamide is a monounsaturated primary fatty acid amide synthesized from palmitoleic acid and ammonia [45].

Note: Compounds are reported as putatively annotated based on accurate mass and matching against the Agilent MassHunter METLIN Metabolomics Database (matching score ≥ 90%, mass error ≤ 5 ppm). No authentic standards or MS/MS fragmentation were used for confirmation. Therefore, these annotations correspond to Metabolomics Standards Initiative Level 2 of identification.

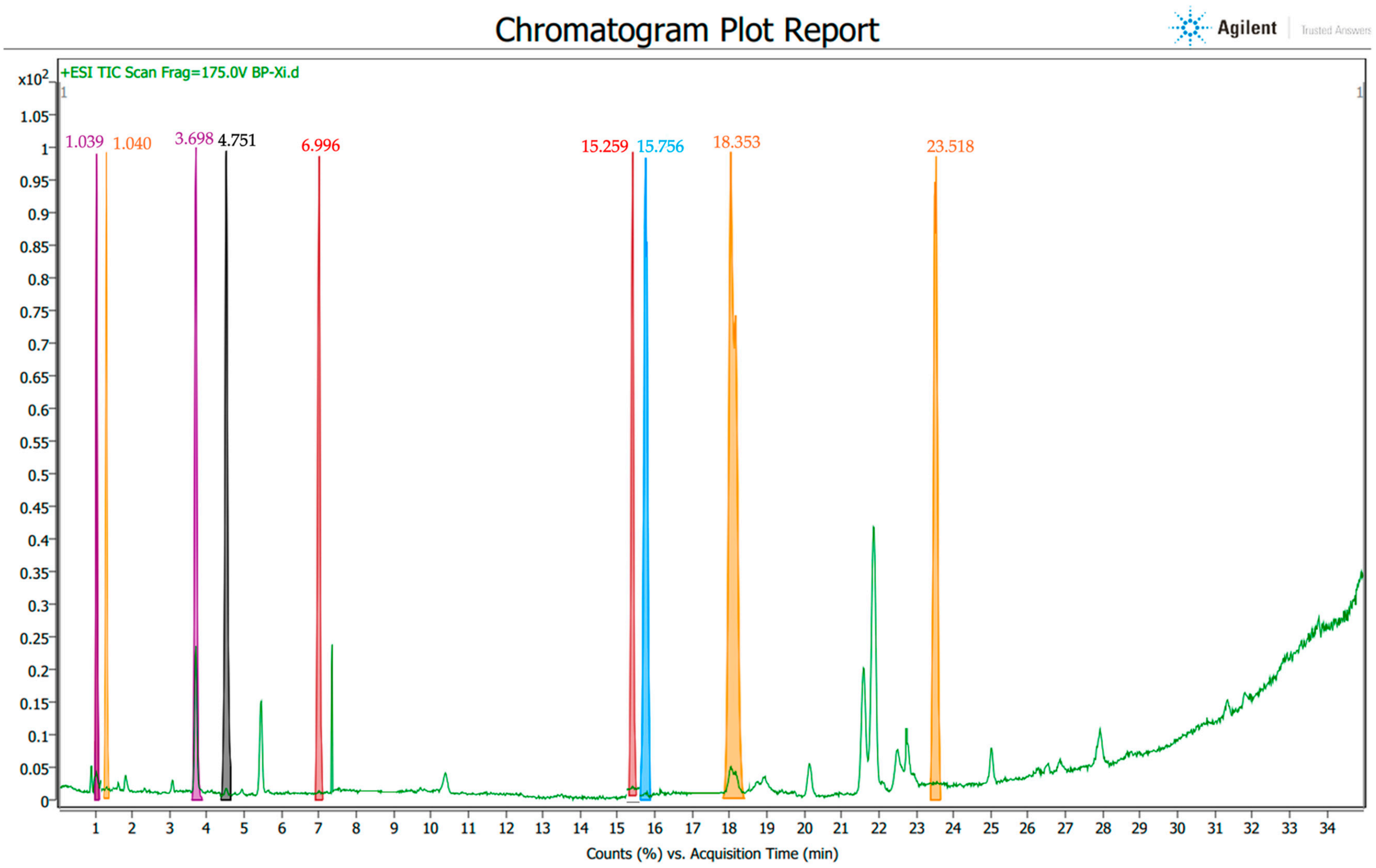

Chromatogram Plot Report
Agilent
Trusted Answers
+ESI TIC Scan Frag=175.0V BP-Xi.d
x10^2
1.039
1.040
3.698
4.751
6.996
15.259
15.756
18.353
23.518
Counts (%) vs. Acquisition Time (min)

a)

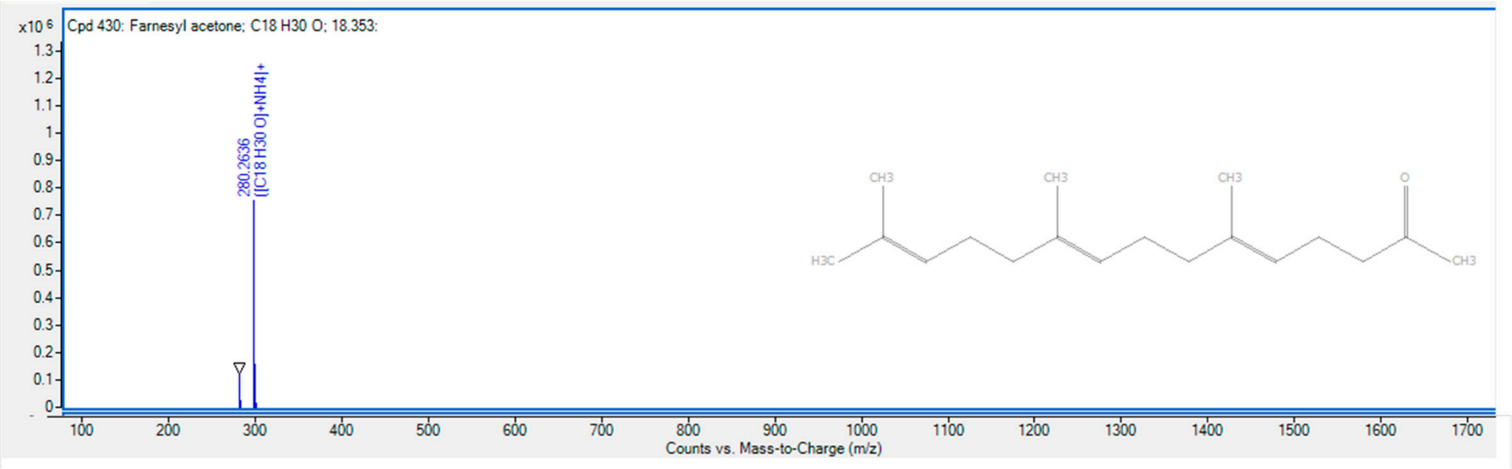

Cpd 430: Farnesyl acetone; C18 H30 O; 18.353:
x10 6
280.2636
([C18 H30 O]+NH4]+
CH3
CH3
CH3
O
H3C
CH3
Counts vs. Mass-to-Charge (m/z)

b)

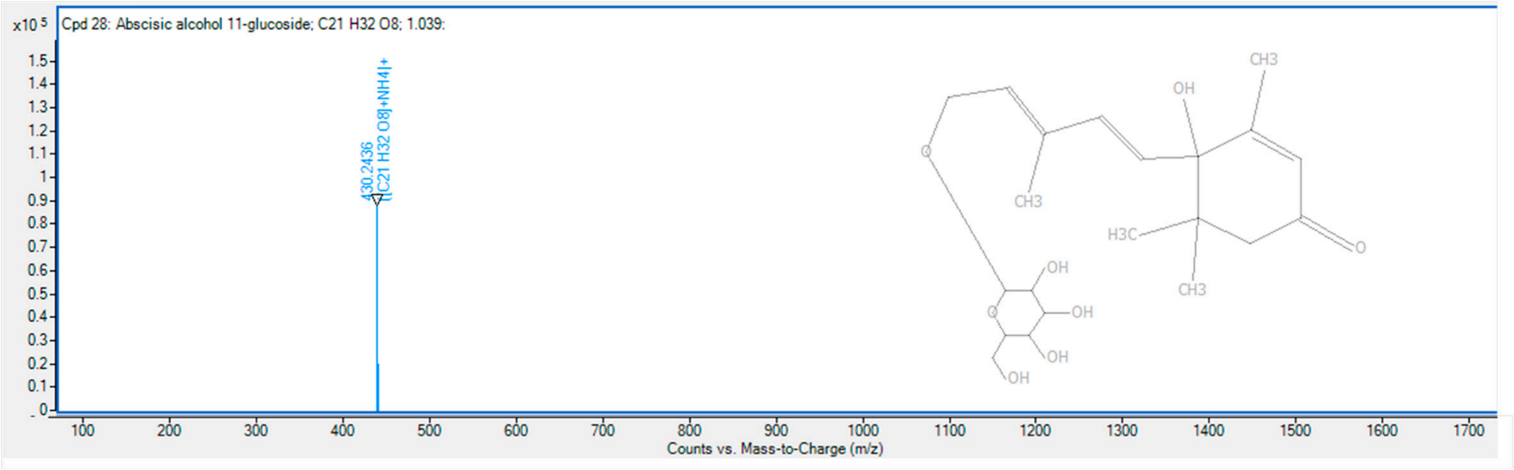

Cpd 28: Abscisic alcohol 11-glucoside; C21 H32 O8; 1.039:
x10 5
430.2436
([C21 H32 O8]+NH4]+
CH3
OH
CH3
H3C
CH3
O
OH
OH
OH
OH
Counts vs. Mass-to-Charge (m/z)

c)

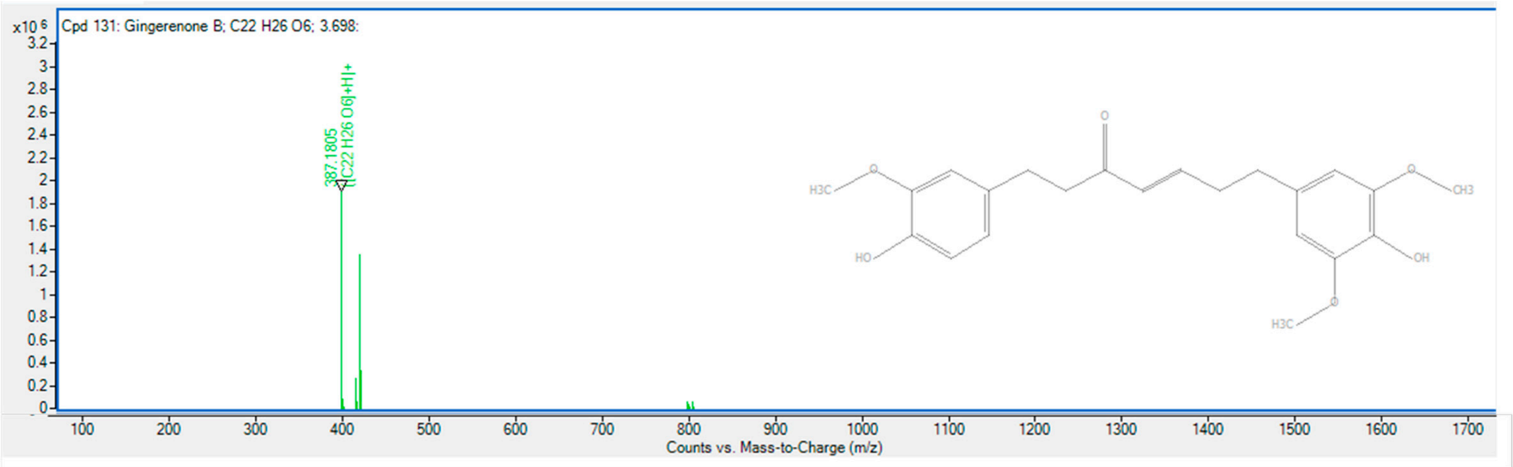
Cpd 131: Gingerenone B; C22 H26 O6; 3.698:
x10 6
387.1805
([C22 H26 O6]+H]+
Counts vs. Mass-to-Charge (m/z)
H3C
HO
O
CH3
OH
H3C

d)

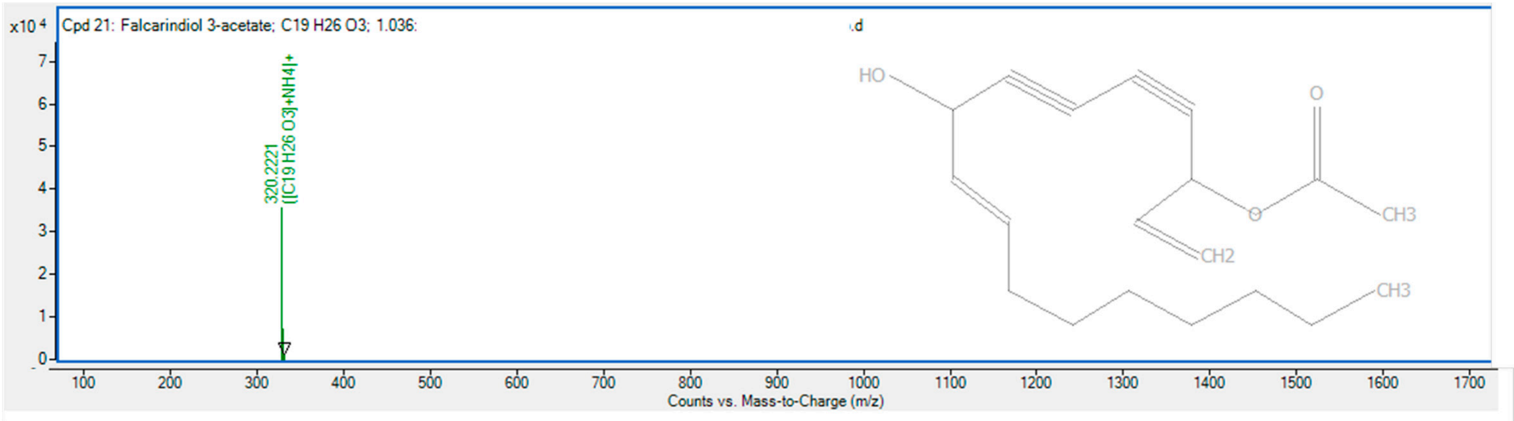
Cpd 21: Falcarindiol 3-acetate; C19 H26 O3; 1.036:
.d
x10 4
320.2221
([C19 H26 O3]+NH4]+
Counts vs. Mass-to-Charge (m/z)
HO
O
CH3
CH2
CH3

e)

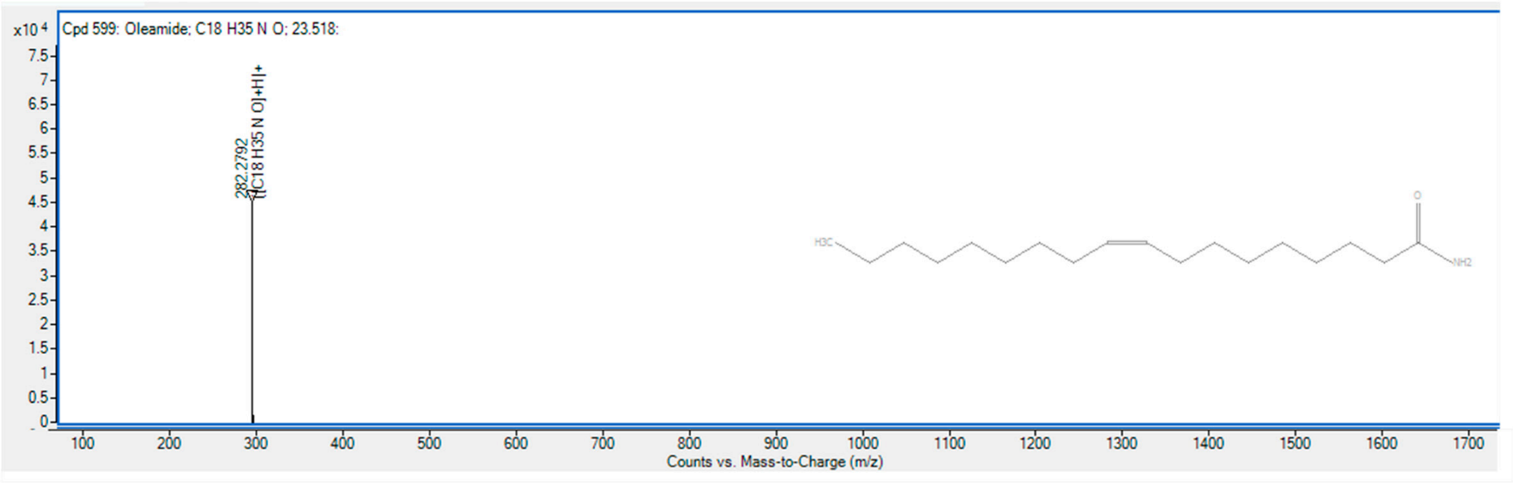

Cpd 599: Oleamide; C18 H35 N O; 23.518:
282.2792
([C18 H35 N O]+H]+
Counts vs. Mass-to-Charge (m/z)

f)

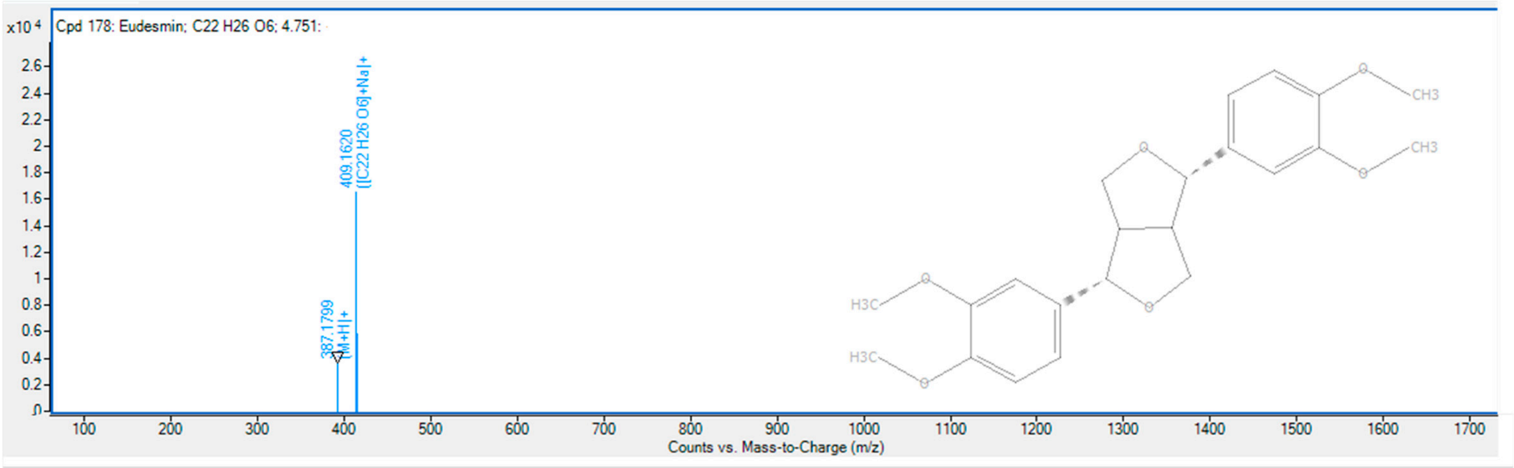

Cpd 178: Eudesmin; C22 H26 O6; 4.751:
409.1620
([C22 H26 O6]+Na]+
387.1799
[M+H]+
Counts vs. Mass-to-Charge (m/z)

g)

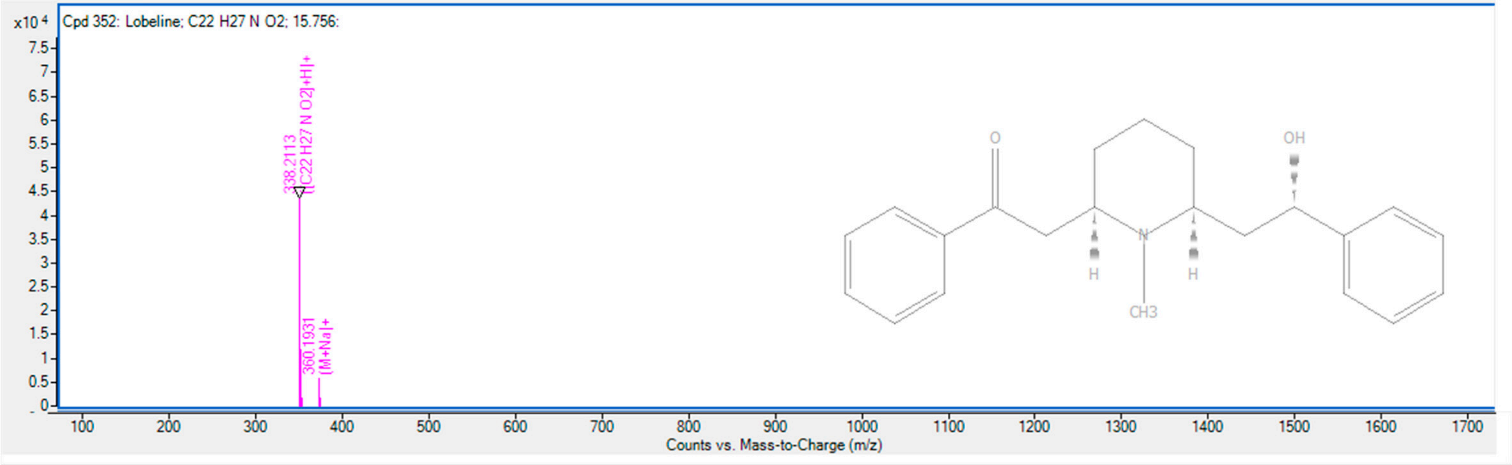

Cpd 352: Lobeline; C22 H27 N O2; 15.756:
x10 4
338.2113
([C22 H27 N O2]+H)+
360.1931
(M+Na)+
Counts vs. Mass-to-Charge (m/z)
O
OH
N
H
H
CH3

h)

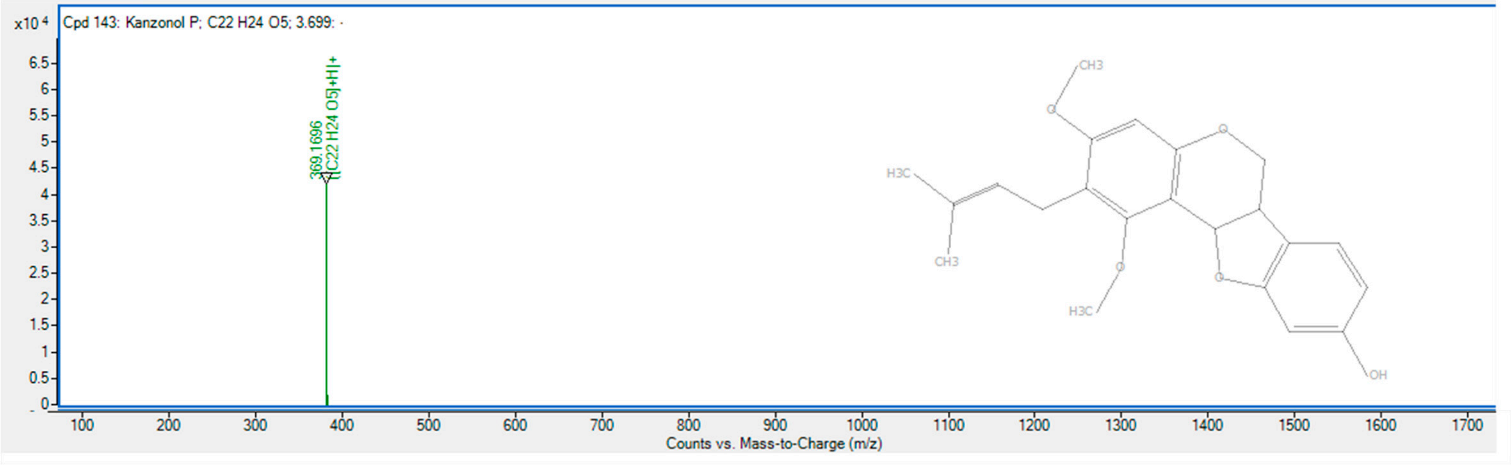

Cpd 143: Kanzonol P; C22 H24 O5; 3.699: -
x10 4
369.1696
([C22 H24 O5]+H)+
Counts vs. Mass-to-Charge (m/z)
CH3
O
H3C
CH3
H3C
OH

i)

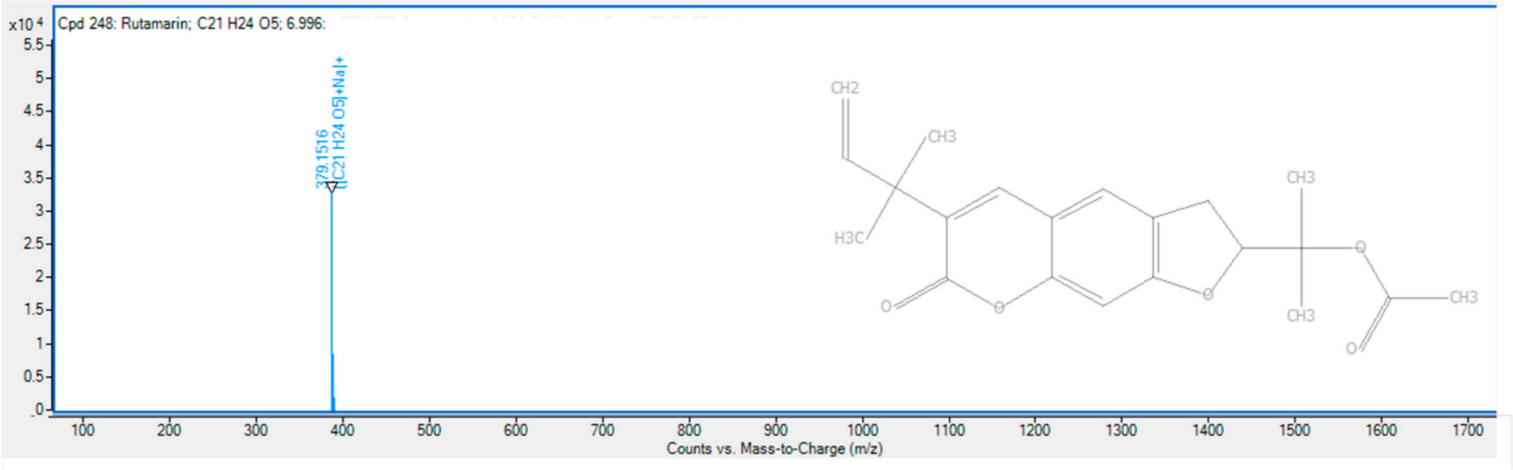

j)

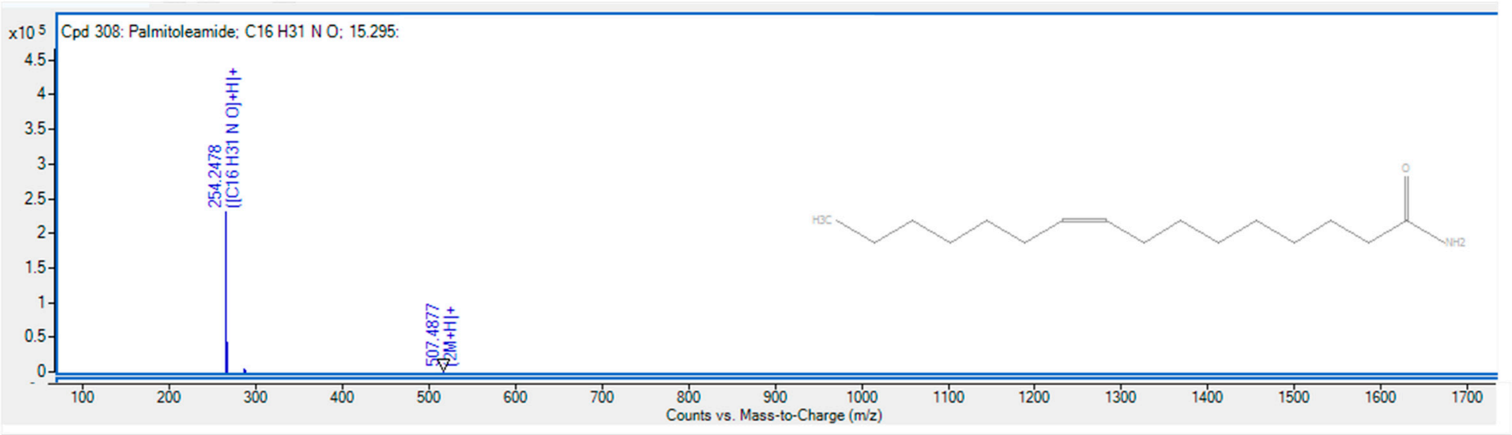

k)

Figure S2. LC-QToF-MS chromatographic profile and extracted ion chromatograms of the top ten putatively annotated compounds in *Xyris* spp. bee pollen extract ranked by database matching scores in positive ESI mode. Panel (a) shows the overall LC-QToF-MS chromatogram across the full retention time range, while panels (b–k) show the extracted ion chromatograms corresponding to the compounds listed in Table S2.
